# Evaluating Contaminant Effects and Blend Ratios on the Alkaline Hydrolysis of Polyester Textile Streams

**DOI:** 10.1021/acssusresmgt.5c00302

**Published:** 2025-09-05

**Authors:** Charlotte M. Wentz, Maxwell D. Mevorah, Allison Carranza, McKenzie L. Coughlin, Amy Engelbrecht-Wiggans, Thomas P. Forbes, Zois Tsinas, Amanda L. Forster

**Affiliations:** † Material Measurement Laboratory, 10833 National Institute of Standards and Technology, Gaithersburg, Maryland 20899, United States; ‡ Chemistry and Biochemistry Department, 1068 University of Maryland, College Park, College Park, Maryland 20742, United States; § Chemistry and Biochemistry Department, 5631 West Virginia University, Morgantown, Virginia 26506, United States; ∥ Mechanical Engineering Department, Rochester Institute of Technology, Rochester, New York 14623, United States; ⊥ Theiss Research, La Jolla, California 92037, United States

**Keywords:** chemical recycling, textiles, cotton reuse, hydrolysis, waste management, post-consumer

## Abstract

The increasing amounts of discarded textiles represent a potentially valuable resource that could be reclaimed, for example, by chemical techniques. This work underscores the significance of utilizing chemical recycling techniques for multicomponent fabrics under mild reaction conditions to investigate the reusability of recovered components. We present a method for recovery of cotton, elastane, and nylon from polyester blends through mild alkaline hydrolysis supported with a phase-transfer catalyst. To juxtapose the impact of these various fibers on the depolymerization of the polyester component into terephthalic acid (TPA), consistent reaction conditions were maintained. The average TPA yield (by mass) was 93.9 ± 2.8% for pre-consumer materials and 89.5 ± 3.1% for post-consumer materials. This comparative analysis provides insights into factors contributing to the observed decrease in the TPA yield. Inimitable to this study, an analysis of the reuse potential of recovered cotton via tensile strength was performed. The average cotton recovery (by mass) was 95.9 ± 0.8%. Comprehensive material characterization of all recovered components was performed. This research paves the way for a deeper understanding of the potential contamination of TPA, the quality of recollected fibers, and what components of a mixed textile stream act as potential “disruptors” to recyclability.

## Graphical abstract

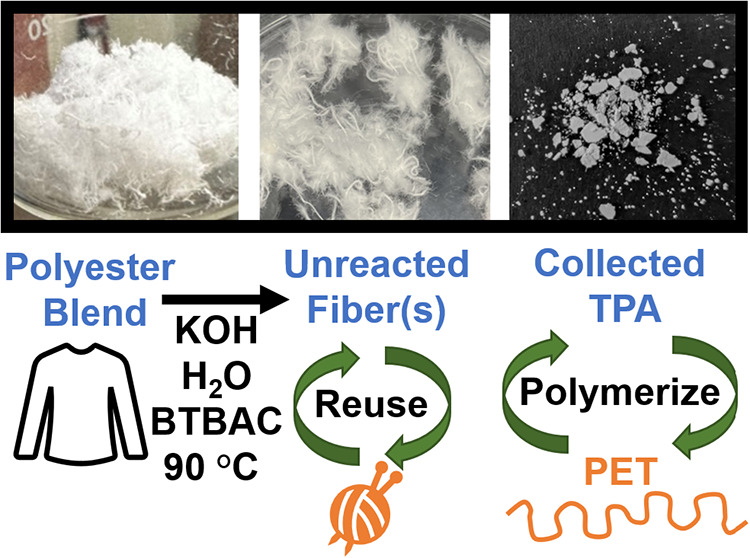

## Introduction

Recent studies of consumer waste have shown that the volume of textiles being discarded in the U.S. is growing, and there is increasing interest in reclaiming this material.^1^ The complex nature of textile waste, encompassing a diverse range of fibers from natural to synthetic, and the prevalence of blended fabrics, all present challenges when reclaiming textile waste.^1,2^ Mechanical recycling, while cost-effective, exhibits limitations when processing multi-fiber blends, particularly those containing elastomeric fibers, and yields low-value products.^3–7^ Less than 1% of textiles are recycled back to fibers.^8,9^ Chemical recycling offers a promising alternative for maximizing resource recovery.^10,11^

Previously, studies have explored polyester fiber depolymerization into reusable monomers like terephthalic acid (TPA) and bishydroxyethyl terephthalate (BHET) (see Table S1 for an abbreviation glossary).^12–15^ Common depolymerization methods include glycolysis^3,16–18^ and hydrolysis.^19–24^ Dissolution, which is effective for fiber separation but does not facilitate depolymerization, is not considered here. Existing studies have encountered challenges, including the degradation of other synthetic fibers and cotton, resulting in the need for additional purification procedures, limiting reuse potential.^20,25^ Chemical recycling systems preferentially utilize pre-consumer textile waste (e.g., excess cuts, unused textiles) to minimize issues related to variable content (known textile construct, avoids dyes, additives, etc.). The majority of textile waste, however, is post-consumer materials, which are only beginning to be investigated in chemical recycling studies.

This study investigates the depolymerization of polyester-based textile blends into TPA utilizing mild alkaline hydrolysis, such as polycotton (P:C) and elastane blends. The compare–contrast design of this investigation between well-known textile blends and unknown post-consumer textiles enables hypotheses regarding conditions (dyes, additives, fiber types, blend percentage, inaccurate labels, or elastomeric fiber constructs) that affect the reuse potential of recovered fibers and TPA. Our findings show an average TPA yield (by mass) of 93.9 ± 2.8% for pre-consumer materials and 89.5 ± 3.1% for post-consumer materials. Since resource collection of recycled cotton holds the highest potential for fiber-to-fiber recycling, key parameters for evaluating the suitability for re-spinning include microstructural changes, fiber length, and tensile strength.^26^ Others have studied parameters important to the reuse of collected cotton,^5,18–21^ but solid recollected fibers (excluding cotton) post-reaction are vastly overlooked. This study thoroughly examines reclaimed cotton for all of these parameters for reuse, including the often-overlooked tensile strength. Given cotton’s resource-intensive nature^27^ and increasing demand, recycled cotton fibers hold precedence for being a focus for fiber recollection.^28^ Currently, only 1% of the cotton market comprises recycled cotton, primarily from mechanical recycling of pre-consumer fabrics.^27^ This research offers a deeper understanding of the potential contamination in both TPA and recollected fibers, specifically identifying textile stream components that act as potential “disruptors” to recyclability.

## Materials and Methods

This work utilizes alkaline hydrolysis with phase-transfer catalysis (PTC) to enhance TPA yield^19^ across a 4 h reaction, ensuring 100% yield. We maintain consistent reaction parameters, 10 wt % KOH, and 0.2 wt % benzyltributylammonium chloride (BTBAC) at 90 °C across all material types. Figure 1 illustrates this approach and shows the materials pre- and post-reaction. Various polyester (P) blends were laboratory-made with known pre-consumer fibers, including cotton (C), elastane (L), and nylon (N), to contrast post-consumer samples (Table S2). Specifically, lab-made blends of 100:0, 75:25, 50:50, and 25:75 P:C were created. Other pre-consumer woven textile blends investigated include 88:12 P:L, 92:8 C:L, and lab-made blends of 80:20, 50:50, 25:75 P:(N:L). Post-consumer comparisons utilized a 52:48 P:C T-shirt and a 0:100 P:C pair of pants. Lastly, buttons or zippers, often composed of commodity plastics, are considered disruptors;^29^ thus, a post-consumer zipper was tested. Each material type was processed five times (*n* = 5) to assess reproducibility. Additional details about materials and methods can be found in the Supporting Information (SI).

**Figure 1:**
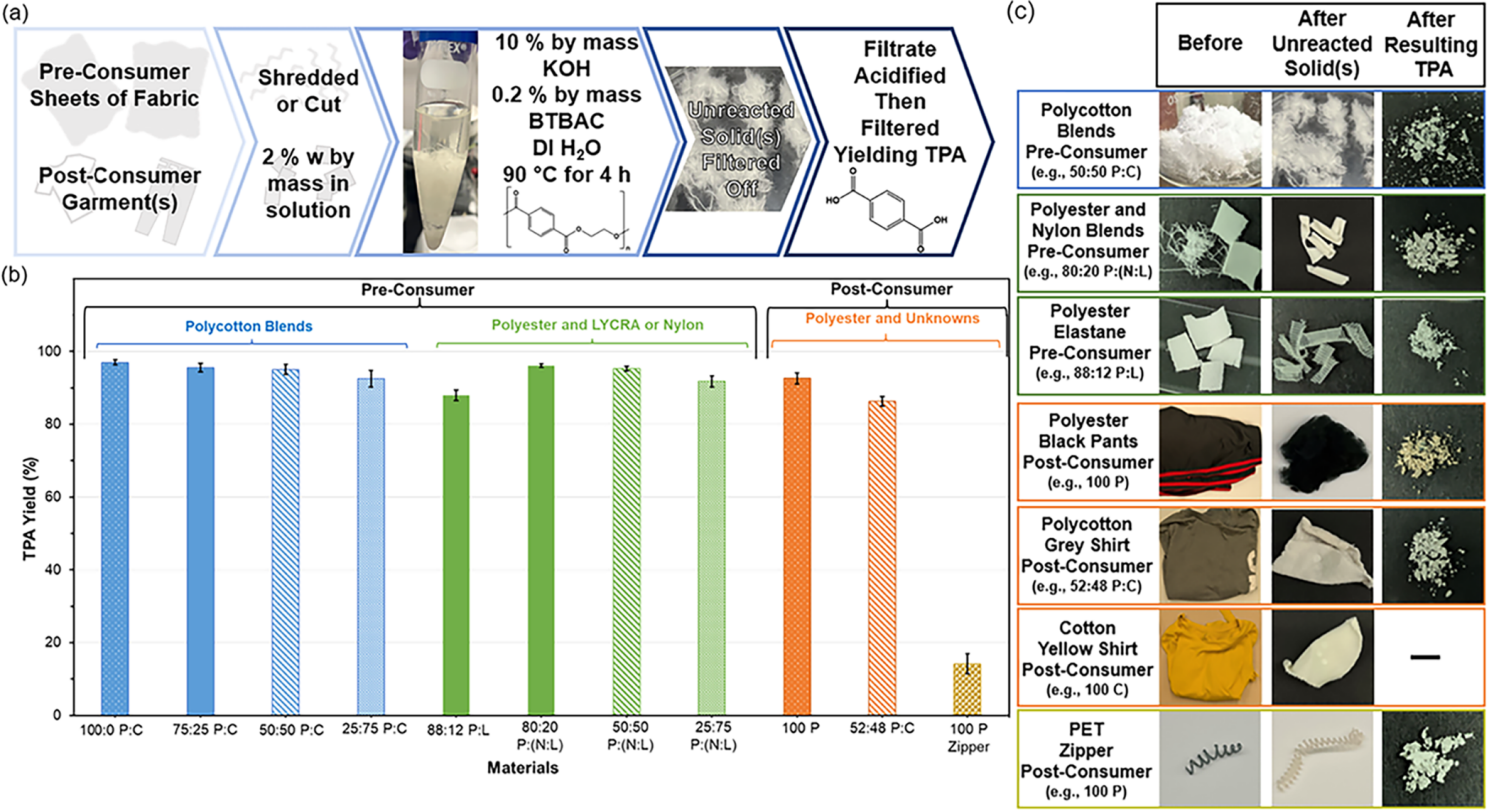
Depolymerization of polyester-containing textile blends. (a) Schematic pathway. (b) Average TPA yields and corresponding standard deviations. (c) Images of the primary material scope.

Assessing the reuse potential of all recovery materials was critical to this study. For TPA, trace contaminants from dyes, additives, or other textile components were identified with nuclear magnetic resonance (NMR), fourier transform infrared spectroscopy (FTIR (Mid-IR)), thermogravimetric analysis (TGA), and pyrolysis gas chromatography mass spectrometry **(**Py-GC-MS) (see the SI for instrument specifications). Collected solid fibers (C, N, L) were analyzed utilizing all of the above, plus scanning electron microscopy (SEM) and near-infrared spectroscopy (NIR). For cotton fibers, quality was assessed by mass recovery, surface chemistry changes (FTIR), degradation temperature changes (TGA), and fiber strength through tensile testing. Differential scanning calorimetry (DSC) was also employed for the thermal property analysis.

## Results and Discussion: Pre-Consumer Blends

### Polycotton (P:C) Blends

We performed workshops and discussions with chemical recycling industry partners, where we identified their scaling challenges.^29,30^ Many chemical recyclers and sorters of textiles emphasized that their recycling systems primarily accept polyester fibers (>80%) and are reluctant to accept other synthetic or elastomeric fibers. This led to inquiries about mixed feedstocks such as whether cotton fibers could hinder the depolymerization of polyester, other fiber types might degrade and contaminate recovered small molecules, current systems prioritize maximizing small molecules yields due to an established market for recycled TPA but limited infrastructure for recycled cotton, and polyester content below 80% may be unreliable and contain other materials of interest that impede recycling or make cost-extraction unprofitable. These feedstock limitations could hinder the development of more inclusive textile recycling systems.^29,30^

Specific TPA yields for each material class are listed in Figure 1b. In short, all P:C blends had an average TPA yield (by mass) of 95.0 ± 1.6%, with an average percent recovery of the unreacted solid cotton fibers of 95.9 ± 0.6%. Figure 2c shows materials before and after reaction, including recollected TPA and solid fibers. No notable difference in TPA or cotton yield was observed with changes in P:C blend ratio. Collected TPA identity is confirmed via FTIR, as shown in Figure 2a, with spectra closely matching commercially available TPA. TGA revealed that the recovered TPA exhibited a thermal degradation profile similar to that of distributor TPA, with a characteristic lower degradation temperature than the parent polyester fibers, Figure 2b. Proton NMR of polyester fibers and TPA showed no unidentifiable peaks, all polyester carbon signals were lost, and all ^1^H NMR peaks expected for the aromatic ring in TPA were present.

**Figure 2:**
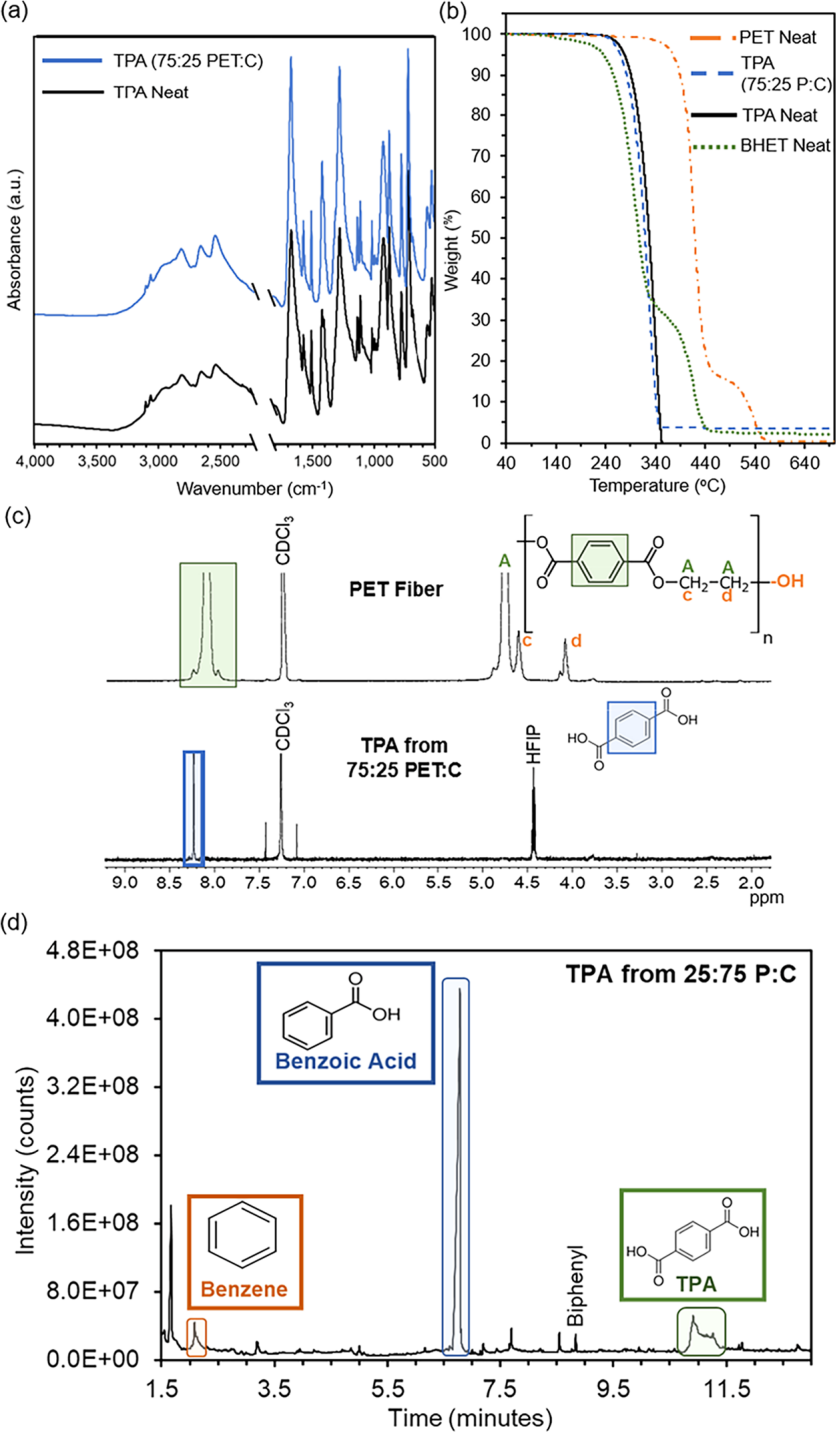
Characterization of collected TPA with (a) FTIR, (b) TGA, (c) NMR, and (d) Py-GC-MS chromatogram.

Pyrolysis GC-MS detected polymeric materials, such as unreacted fiber contaminants or oligomeric units from partially reacted fibers, in the recovered TPA. Mass spectra from major chromatographic peaks were identified against the NIST MS library, revealing key TPA components and no trace of cotton or polyester components, Figure 2d. This instrument also analyzed solid polyester (Figure S1) and cotton fibers (Figure S2). Overall, this alkaline hydrolysis of P:C textiles yields high TPA, unaffected by the polyester-to-cotton ratio. Moreover, this series of materials addressed curiosities regarding acceptable feedstock for the chemical recycling of polyester fiber blends.

### Characterization of Cotton

For re-spinning into yarns, cotton fibers must be >13 mm long and have high strength (about a minimum of 3.0 g per denier or about 0.038 N) and appropriate fineness (fiber diameter). These properties directly influence spinning efficiency and yarn quality.^26^ This study emphasized fiber strength, a critical parameter for re-spinning, via single fiber tensile testing, which is often undocumented in other studies.

Molecular spectroscopies showed no chemical changes or mercerization in cotton pre- or post-alkaline hydrolysis, Figure 3a,3b. Mercerization can change cotton’s strength, luster, absorbance, and dye affinity. Mid-IR can indicate the cellulose I to II transformation in the 3600 to 2800 cm^–1^ region; cellulose II shows a broader, slightly shifted peak to a lower wavenumber. While studies show that NaOH concentrations of 10 wt % or more can mercerize cotton,^31^ this study used 10 wt % of KOH, as it is less harsh due to its weaker interactions with cotton.^32^ Studies on bacterial cellulose demonstrate that KOH treatment can result in less fiber shrinkage compared to NaOH treatment.^33^ The recycled cotton’s FTIR spectrum closely resembled the cotton pre-reaction, suggesting minimal to no mercerization.

**Figure 3:**
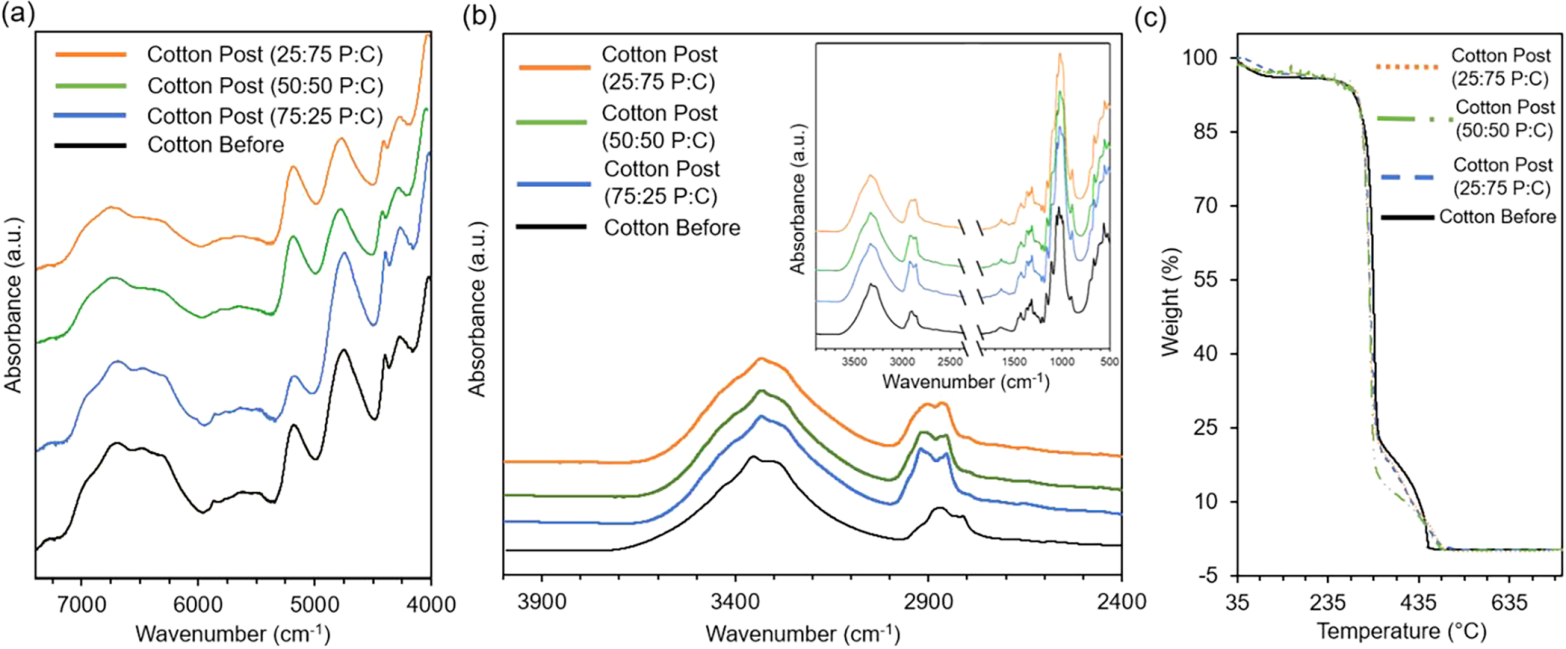
Characterization of cotton fibers with (a) NIR spectra, (b) MID-IR spectra (full spectra inlet), and (c) TGA.

Thermal analysis elucidated molar mass or crystallinity changes in fibers after alkaline hydrolysis. TGA showed no loss of cotton fiber integrity, exhibiting the same degradation temperature pre- and post-reaction, Figure 3c. Regardless of the P:C ratios, no discernible differences were found between recovered cotton and neat (pre-reaction) cotton. Polyester fibers had an onset degradation temperature of 414 °C (Figure S8) while cotton fibers had 361 °C (Figure S9), allowing detection of any remaining polyester fibers. While DSC provided no useful information on cotton fibers, we observed thermal properties with a melting point (*T*_m_) of 251 °C, glass transition temperature of 78.4 °C, and percent crystallinity of 55.0% (assuming the enthalpy of fusion for 100% crystalline polyethylene terephthalate (PET) is 140 J/g) (Figure S11).

SEM examined the fiber surface microstructure and measured the diameter change. Figure 4a shows pre-consumer cotton fibers with a line-like pattern and smooth twisted surface, typical morphology, averaging about 16.5 ± 0.5 μm in diameter. Post-reaction, Figure 4b, no significant changes in surface texture or diameter were observed, 16.6 ± 0.6 μm. Mercerized cotton fibers typically exhibit a swollen and roughened texture due to the microfibril reorientation.^31^ The absence of these morphological changes further supports the limited effect of the reaction conditions on fiber integrity.

**Figure 4:**
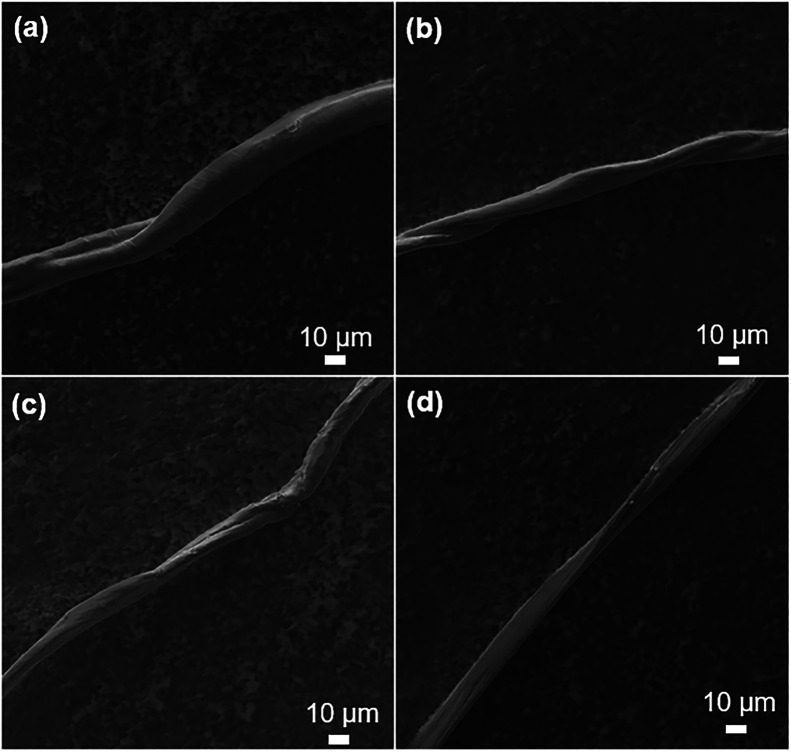
Representative SEM images of (a) 100% cotton fibers pre- and (b) post-reaction. SEM of post-consumer 100% cotton yellow shirt fiber (c) pre-, and (d) post-reaction.

Bulk fiber strength was analyzed through tension testing. The Weibull scale parameter (a measure of population failure time)^34^ decreased slightly, but not significantly, from 0.046 to 0.037 N for pure cotton and 0.038 N for the 25:75 P:C, Figure 5. Comparing pre- and post-reaction cotton failure distributions, a two-sample Kolmogorov–Smirnov (KS) test yielded *p*-values of 0.022 (0:100 P:C) and 0.064 (25:75 P:C). More information about confidence intervals is in Table S3. Post-reaction cotton results are shifted to the left of the neat cotton, consistent with the KS test, indicating a slightly larger drop for pure cotton, but this drop was much smaller than the inherent sample variability.

**Figure 5:**
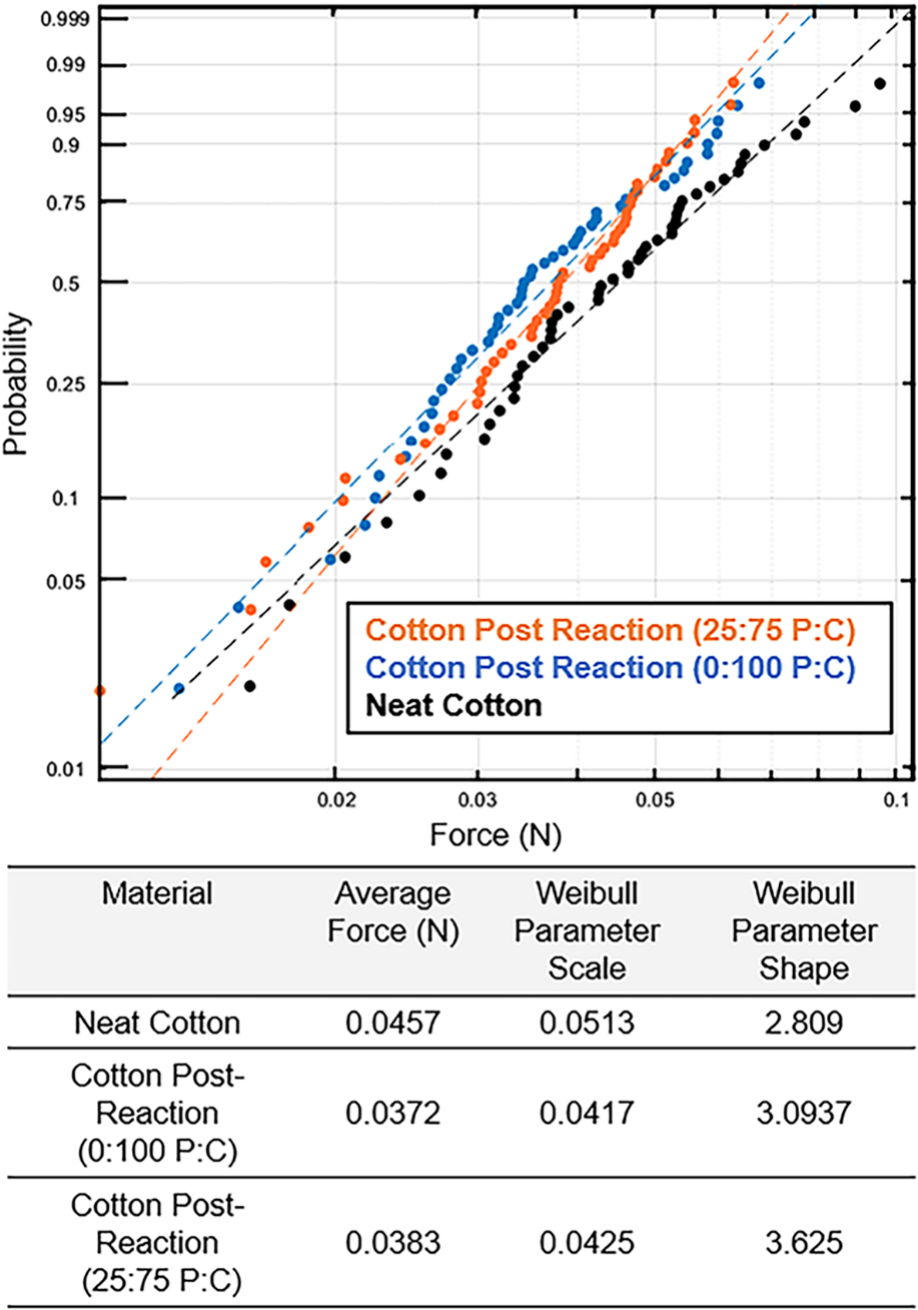
Single fiber tensile testing Weibull plots of pre-consumer cotton fibers pre- (black) and post-reaction (blue or orange) with average force and Weibull parameters in the table below the graph.

The Weibull shape parameter (which describes how failure rates change over time)^34^ appeared unchanged by the reaction process; data for the three conditions were roughly parallel. If anything, the shape parameter increased post-reaction, indicating less variability after recycling. The shape parameter, linked to the inherent flaw distribution, typically modeled as a Poisson random variable, suggests no new flaws were introduced since it remained constant. Tension tests are significant to the cotton’s reusability and are yet to be documented in other publications.

### Polyester and Other Fiber Blends (P:(N:L))

Mechanical recycling, often touted as closed-loop, is limited by its inability to process textiles containing elastane or high proportions of synthetic fibers like nylon.^35^ Chemical recycling could reclaim these materials, but certain chemical recycling processes, particularly those involving high temperature, strong alkaline or acidic conditions, or specific catalysts, can degrade elastomeric fibers, requiring additional purification. Previous studies reported partial nylon degradation (38.4% of the fibers hydrolyzing at 50 wt % NaOH).^36^ More recently, Andini et al. utilized glycolysis to depolymerize polyester fibers in the presence of nylon and elastane, resulting in significant mass loss and requiring additional purification to remove the other small molecules from BHET.^18^ In contrast, catalyst-assisted alkaline hydrolysis, demonstrated by Wu et al., shows promise in preserving elastomeric and cotton fiber integrity with minimal nylon degradation.^37^ This work characterizes a more non-destructive method of retrieving these fibers from mixed polyester textiles via depolymerization under mild alkaline conditions.

The same neat L within the P:L sample could not be obtained for testing; a similar L sample was used for the neat comparisons in TGA (see SI). Thermal analysis suggested any remaining unreacted polyester fibers were undetectable, indicating full separation. Figure 6a, TGA, indicated the thermal stability of the reclaimed elastane fibers, with a similar profile to that of a neat elastane sample. DSC revealed a *T*_m_ corresponding to the elastane component of about 17.8 °C, while the polyester fiber’s *T*_m_ was not observed, suggesting successful depolymerization. Figure S12 depicts the original profile of the P:L material, where both *T*_m_ are observed.

**Figure 6:**
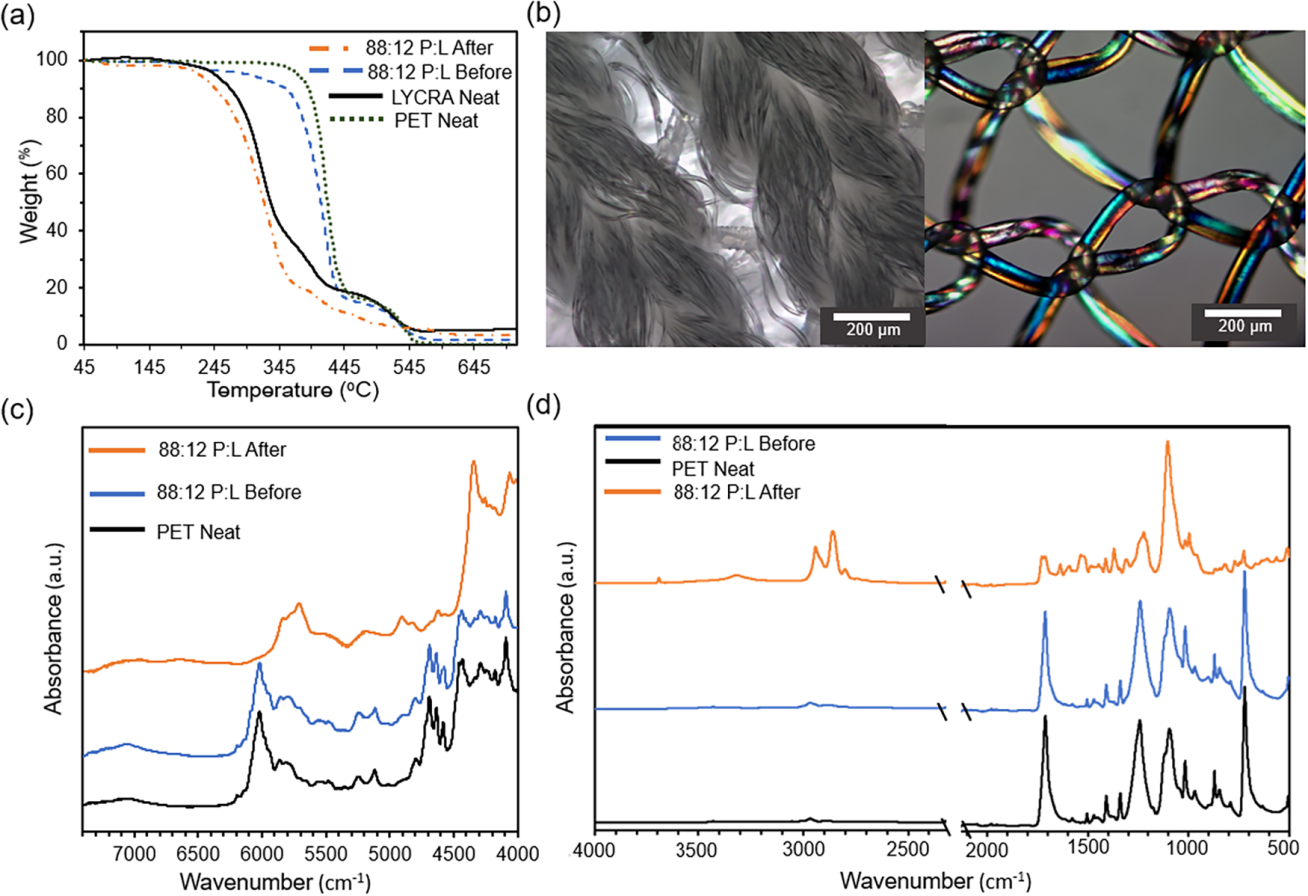
Characterization of pre-consumer materials with (a) TGA, (b) P:L pre- (left) and post-reaction under polarized light (right), (c) NIR spectra, and (d) FTIR Mid-IR spectra.

Both visual inspection (Figure 1c) and optical microscopy (Figure 6b) show retention of elastane fiber structure from 88:12 P:L, with minimal evidence of unreacted P fibers or residue. However, lower-yielding experiments show frayed fibers, believed to be residual polyester, under optical microscope and SEM (Figures S16–S23), due to their smaller diameter than elastane. Additional SEM images of the 88:12 P:L fibers are given in the SI. Further molecular spectroscopy, Figure 6c,6d, confirmed recovered fibers as elastane with no detectable polyester signatures for high yielding experiments. Polyester signatures are easily detectable in P:C blends up to a 5:95 P:C ratio. Note that the IR beam cannot penetrate to interrogate the elastane core, so it was not detectable.

These findings suggest that elastomeric fibers within the fabric matrix may reduce the fiber availability for depolymerization under the studied conditions due to the wrap-and-woven construct, sterically hindered polyester fibers in crevices. Recovered elastane fibers show promise for reuse in applications requiring stretch or elasticity, such as new fabrics and packing materials.

For separate sheets of 81:19 N:L blended with polyester, recovered solid fibers exhibited a curled appearance, Figure 1c, but otherwise retained their original characteristics. Both FTIR and NIR spectroscopy confirmed integrity, with spectra closely resembling those of the original neat nylon and elastane fibers, as shown in Figure 7a,7b. The TGA exhibited a slight decrease in thermal stability of the recovered fibers compared to the pre-reaction material, Figure 7c. DSC of the recovered 81:19 N:L juxtaposed to the original N:L sheets showed a slight decrease in *T*_m_ for the nylon and elastane fiber components. Nylon showed an original *T*_m_ of 255.4 °C and a post-reaction *T*_m_ of 241.4 °C. Elastane showed an original *T*_m_ of 22.9 °C, and post-reaction was *T*_m_ of 19.9 °C (Figure S13).

**Figure 7:**
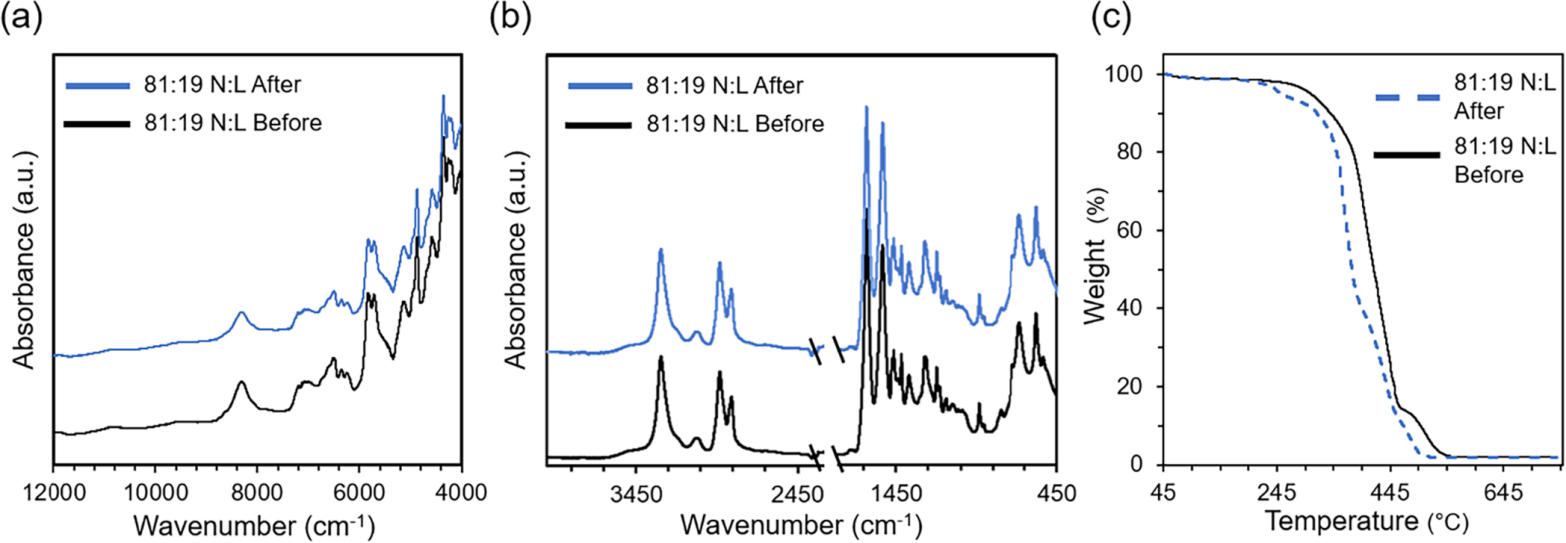
Characterization of pre-consumer materials with (a) NIR spectra, (b) MID-IR spectra, and (c) TGA of N:L fabric pre- (black) and N:L post-reaction (blue).

TPA yield from P:(N:L) blends averaged 94.3 ± 1.9%, with an average percent N:L component recovery of 98.6 ± 1.3%, comparable to P:C blends. However, a notable decrease in TPA yield was observed for the P:L matrix, with an average of 87.9 ± 1.4%. This difference may be attributed to how polyester fibers adhere to elastane during wrapping rather than chemistries or possible reactions, as P:(N:L) materials do not show this trend. Further research into the effects of elastomeric fabric constructs on depolymerization is needed to fully understand this discrepancy.

Due to noticeable TGA curve changes and *T*_m_ shifts in N:L containing samples, a detailed TPA analysis was conducted. Mid-IR spectroscopy confirmed recovered TPA identity (Figure S39a), but it did not reveal L or N effects on TPA recovery. TGA exhibits a similar thermal degradation profile to commercial TPA across all blend ratios (Figure S39b), but with a lower onset temperature by 2 to 3 °C (outside instrumental margin of error), potentially indicating small molecules from L and N fibers passing into the TPA, affecting char formation or adding thermally unstable molecules.

Py-GC-MS analyzed the collected TPA for fiber contaminants or oligomeric units from partially reacted nylon or elastane. TPA from polyester–elastane reactions revealed characteristic fragments corresponding to benzene, benzoic acid, and TPA, as shown in Figure 8a,8b. However, TPA from P­(N:L) blends exhibited additional unidentified peaks between 2 and 6 min above background noise. Similar peaks were observed in solid nylon and elastane fibers (Figures S3 and S4). These peaks may be contaminants from the synthetic fiber processing, as PET and nylon-containing materials are known to release oligomers during washing and drying.^38^ Moreover, solid nylon and elastane fibers run on Py-GC-MS pre-reaction showed peaks aligning with those in collected TPA (Figures S5 and S7). Thus, these unknown components are likely from the original fibers rather than side reaction byproducts. Further research is needed to identify these trace contaminants and assess their impact on recycled TPA and subsequent use.

**Figure 8:**
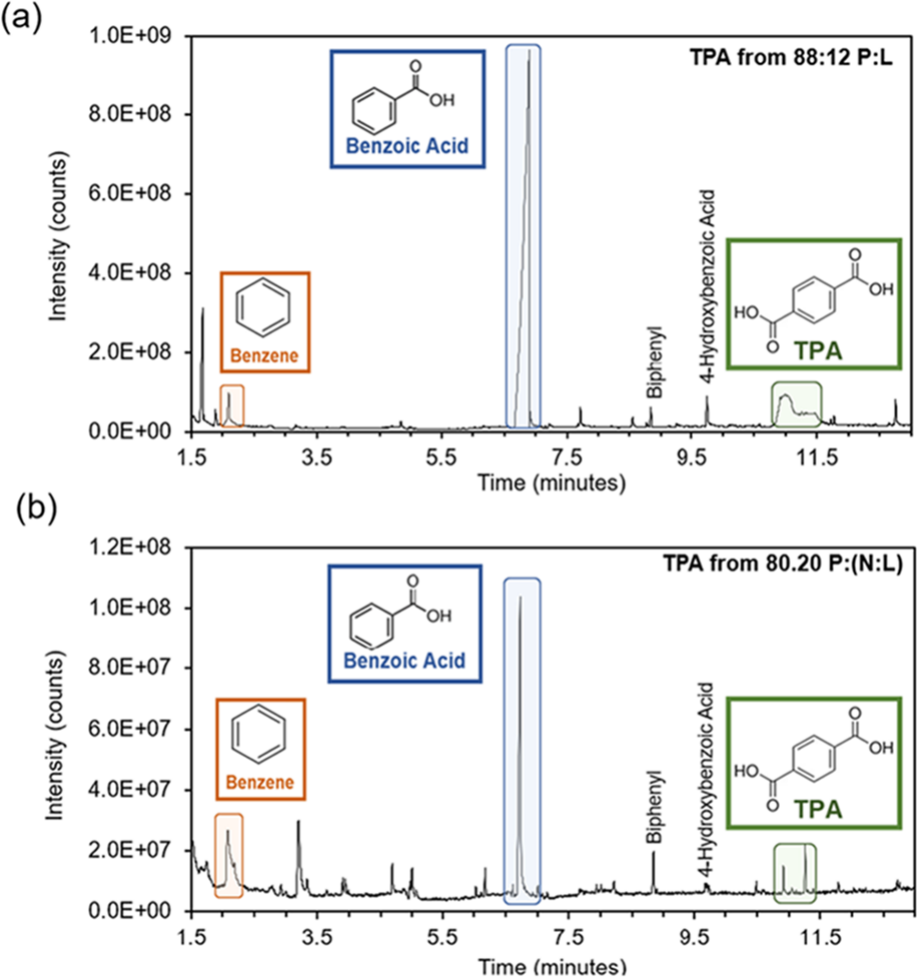
Characterization of TPA produced from pre-consumer materials containing nylon and elastane with (a) GC-MS trace of TPA from P:L experiment, and (b) TPA from P:(N:L) experiment.

For the 92:8 C:L material, our objective was to assess the fiber quality. Briefly, no clear change in cotton–elastane fibers was observed pre- and post-reaction via spectroscopy (Figures S30 and S33), further supporting the lack of cotton mercerization. TGA showed similar onset degradation temperatures pre- and post-reaction (Figure S7). From DSC, the *T*_m_ of the elastane component was found to be 19.0 °C before and 18.4 °C after reaction (Figure S11), indicating that reaction conditions affect some thermal properties of the elastane fibers. An average percent recovery of 98.1 ± 1.0% was obtained.

## Results and Discussion Post-Consumer Blends

### Polyester-Containing Post-Consumer Materials

Two polyester-containing post-consumer textile items were investigated: a 52:48 P:C gray t-shirt with a graphic design and a 100% polyester pair of pants (100:0 P:C) with black and red dyes. These post-consumer materials yielded less TPA than their pre-consumer counterparts. For example, the pre-consumer 100% polyester fibers had an average TPA yield of 97.0 ± 0.7%, whereas the post-consumer 100% polyester pants had an average TPA yield of 92.6 ± 1.5% (4.40% difference). The 52:48 P:C t-shirt had an even wider discrepancy of 8.72%. We hypothesize these lower TPA yields are because of the additional mass of dyes (1 to 5% dye by mass of fiber on average) or finishes, inaccuracies of the stated fiber compositions on garment labels, and/or potential interference from dyes and additives on the depolymerization process. A systematic experiment testing dye interference is recommended to fully understand this discrepancy.

While analysis techniques such as FTIR (Figure S40a) and TGA (S40b) revealed no difference between recovered TPA and distributer TPA, visual inspection showed a noticeable hue change, Figure 1c. While Py-GC-MS analysis might not detect certain dyes (salts), it showed numerous unidentified peaks with high intensity, Figure 9a,9b, potentially unknown fragments, or finishes. Complementary techniques like HPLC or ICP-MS could provide a more comprehensive characterization for future study. For the 52:48 P:C t-shirt, glucose was detected in recovered TPA, likely due to wear and tear exposure, suggesting cotton pieces were not fully removed by filtration. If the reaction conditions broke down cellulose, it would have appeared in Py-GC-MS traces for other TPA specimens from pre-consumer P:C materials. The unreacted cotton fibers from the 52:48 P:C t-shirt lost color and graphics post-reaction. Lastly, the material lost the C=O (1700 cm^–1^) in FTIR (Figure S32) and 8800 cm^–1^ in NIR (Figure S34), further indicating an effective polyester component was removed.

**Figure 9:**
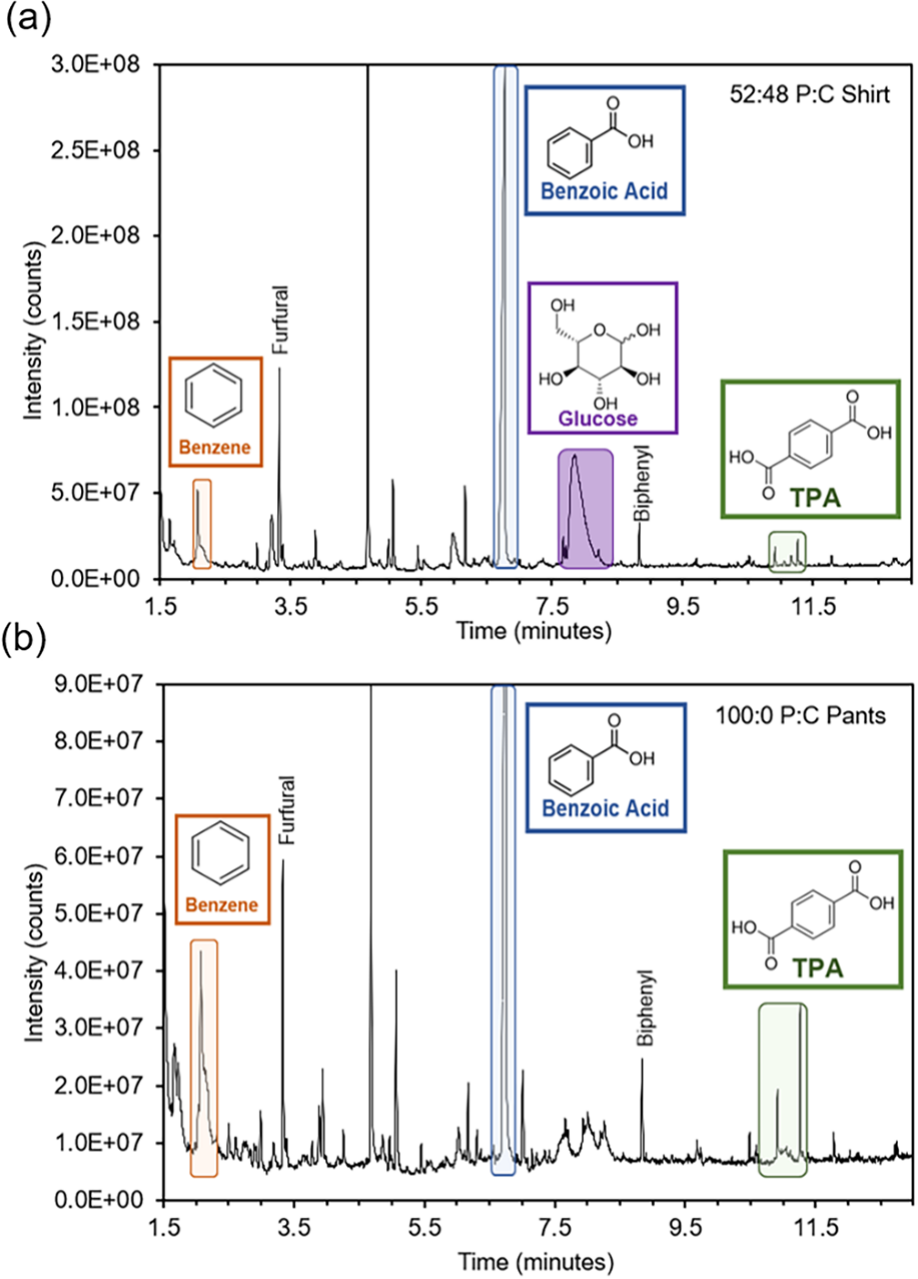
Characterization of post-consumer material’s post-reaction with (a) GC-MS trace of TPA from t-shirt, and (b) GC-MS trace of TPA from 100 P:C pants.

### Cotton Only Post-Consumer Material

A post-consumer 100% yellow cotton shirt was also analyzed. Spectroscopy (Figure S41a) revealed no evidence of mercerization. TGA showed a slight decrease in the cotton fiber onset degradation temperature post-reaction (Figure S41b). SEM, Figure 4c, showed a rougher surface on the post-consumer 100% cotton yellow shirt fibers pre-reaction than the pre-consumer undyed cotton textiles, which may be attributed to dye/detergent deposits or wear. Post-reaction, Figure 4d, surface roughness was reduced, though some small bumps remained. No significant change in fiber diameter was observed between pre-reaction (19.1 ± 1.2 μm) and post-reaction (18.5 ± 1.2 μm) materials. SEM analysis did not show evidence of mercerization of the fibers.

For post-consumer fibers, tensile testing revealed no statistically significant change in tensile strength pre- to post-reaction. A KS test gives a p-value of approximately 0.766, indicating similarity between the two failure distributions, as shown in Figure 10. Neat (pre-reaction) post-consumer cotton shirt and the pre-consumer neat cotton also had no statistically significant difference in failure distribution (KS *p*-value of 0.670, Weibull scale parameters of 0.0510 and 0.0540 N). Different cotton varieties can have different tensile strengths; however, in this case, both feedstocks had statistically similar starting failure distributions. Consistent tensile results pre- and post-reaction for post-consumer fibers suggest this recycled cotton could be successfully reused in spun yarn.

**Figure 10:**
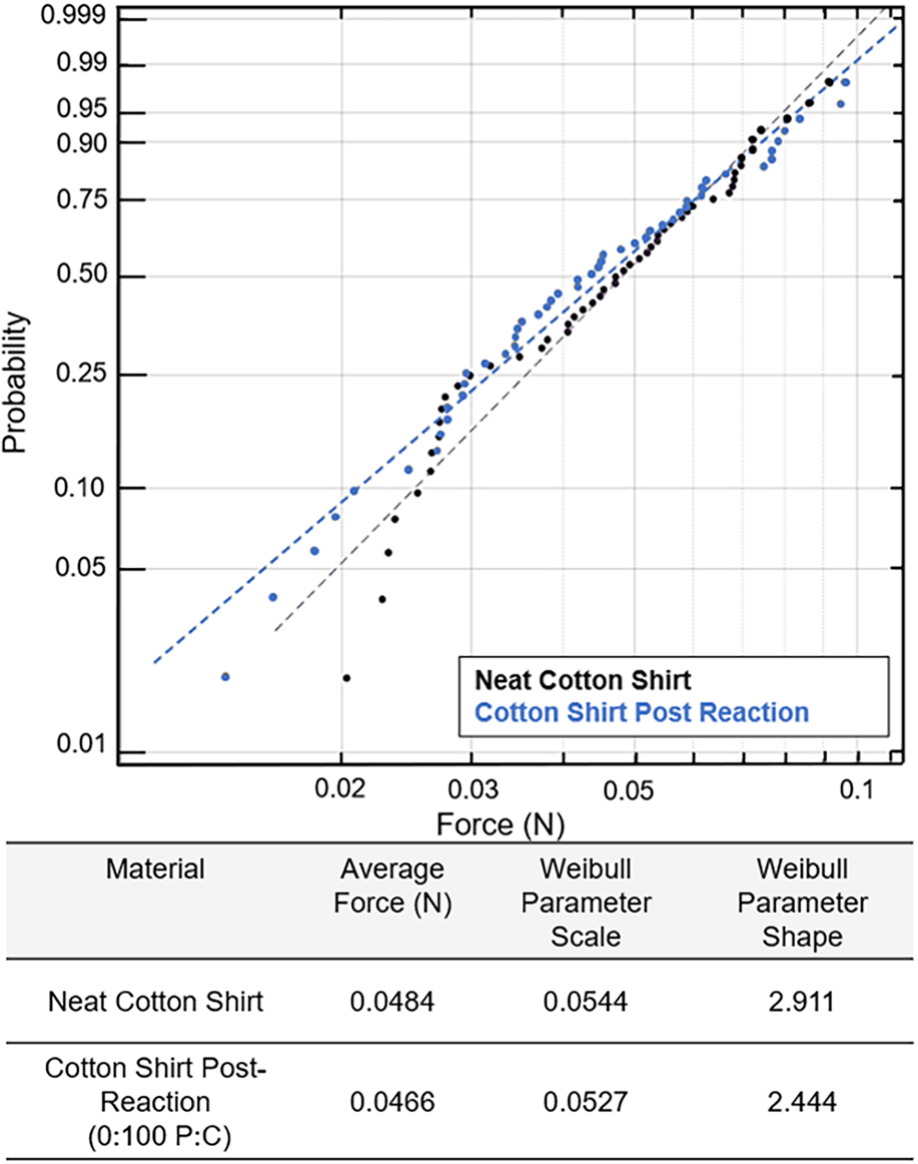
Single fiber tensile testing Weibull plots of post-consumer cotton fibers pre- (black), cotton fiber’s post-reaction (blue or orange) with average force and Weibull parameters in the table below the graph.

### Polyester-Based Zipper

Garments sometimes contain buttons and zippers constructed of PET, polyacetal resin, or other thermoplastic polymer, whose chemical recycling under fiber-depolymerization conditions is not well characterized. Ideally, conditions for fiber depolymerization would also work for buttons and zippers. This study analyzed a zipper primarily made of PET, evidenced by its FTIR (Figure S33), NIR (Figure S37), and DSC (Figure S15). Any other components (such as additives) were undetected. However, the zipper showed a drastic decrease in TPA yield, averaging of 14.2 ± 2.8 %.

DSC traces of the zipper showed a *T*_m_ of 237.0 °C pre- and a *T*_m_ of 239.5 °C post-reaction. Assuming a 100% crystalline PET enthalpy of fusion of 140 J/g, the zipper’s crystallinity changed from 42.3% (before) to 36.25% (after). Cold crystallization temperature also showed a slight decrease and broadening from 211.6 (before) to 197.5 °C (after). This change in PET zipper thermal properties could be attributed to smaller, less perfect crystals formed from depolymerized segments, leading to shorter polymer chains and altered crystallization. Prior studies highlight the importance of molar mass, crystallinity, and additive variations in different PET materials.^39^ For example, bottle-grade PET depolymerizes more readily due to its lower crystallinity.^40^ The crystalline zipper (42.3%) depolymerized slower than similar crystalline polyester fibers (55.0%). NMR can estimate the MW of polyester fiber (7802 g/mol, Figure 2c) versus PET zipper (108,275 g/mol, Figure S26), explaining the lower TPA yield for the zipper. This discrepancy highlights the potential limitations of chemical recycling using milder conditions. Lastly, TPA from the zipper, analyzed by FTIR (Figure S34) and NMR (Figure S31), showed no difference from commercially available TPA and no discoloration.

## Conclusions

This study demonstrates the feasibility of a specific chemical recycling method for mixed textile blends. Mild alkaline hydrolysis, assisted by PTC, selectively depolymerizes polyester fibers from both pre- and post-consumer textile blends. This method consistently achieved high TPA yields for pre-consumer textiles while preserving recovered cotton fiber mechanical strength. Systematically evaluating polyester fiber blend impacts (P:C and P:(N:L)) provides valuable insights into textile garment composition’s relationship with mild alkaline hydrolysis effectiveness. The reusability of both the TPA and fibers from pre-consumer polyester blends was unaffected by fiber percentages.

Pre-consumer materials with elastane and nylon show high TPA purity, with no evidence of nylon or elastane degradation from analytical techniques. Thermal techniques like TGA and DSC showed insightful variations in elastane and nylon thermal properties post-reaction. The impact of these changes on fiber reusability is a future endeavor. Resulting TGA changes in recollected TPA also suggest that elastane and nylon fibers inherently contain unknown small molecules that end up in the TPA.

Increased variability and TPA yield loss were prevalent in post-consumer materials, highlighting potential difficulties in chemical recycling for post-consumer garments. Analytical techniques, Py-GC-MS and TGA, could detect these subtle changes only in the recycled TPA.

Future research is needed to optimize processes for post-consumer materials. Potential partnerships with a fashion brand, providing garments before and after dyes/additive inclusion, could further elucidate their impact on depolymerization and recovered material quality. Additionally, developing dye removal methods, advanced catalysts, or specialized depolymerization conditions could lead to higher-yielding chemical recycling processes for post-consumer mixed textile materials. Exploring reapplication avenues for the recovered elastane and nylon fibers and measurable characteristics impacting their reapplication is another worthy pursuit for future work.

## Supplementary Material



## Data Availability

Available as a NIST Data Repository: doi:10.18434/mds2-3727.

## Notes

GC-MS trace of P neat pre-consumer single fiber; GC-MS trace of C neat pre-consumer single fiber; and abbreviations used in the manuscript and fabric specifications from the manufacture (PDF)

## #

Deceased April 5, 2025.
