# Supplementary material for: Evaluating Contaminant Effects and Blend Ratios on the Alkaline Hydrolysis of Polyester Textile Streams
Source: ACS Sustain Resour Manag. 2025 Sep 5;2(9):1776–85. doi: 10.1021/acssusresmgt.5c00302 (PMC12478850; doi:10.1021/acssusresmgt.5c00302)
Supplement: Supplementary file 1 [file rm5c00302_si_001.pdf]

Electronic Supplementary Information to accompany:

Evaluating Contaminant Effects and Blend Ratios on the Alkaline Hydrolysis of Polyester Textile Streams

Charlotte M. Wentz[1], *Maxwell D. Mevorah*[1,2], *Allison Carranza*[1,3], *McKenzie L. Coughlin*[1], Amy Engelbrecht-Wiggans[4], *Thomas Forbes*[1], *Zois Tsinas*[5,1], *Amanda L. Forster*[*1]

1 Material Measurement Laboratory, National Institute of Standards and Technology, Gaithersburg, Maryland 20899, United States.
2 Chemistry and Biochemistry Department, University of Maryland College Park, MD 20742.
3 Chemistry and Biochemistry Department, West Virginia University, VA, 26506.
4 Mechanical Engineering Department, Rochester Institute of Technology, Rochester, NY, 14623.
5 Theiss Research, La Jolla, California 92037, United States
***Corresponding author:** amanda.forster@nist.gov (240)-586-2650

Section 1: Materials and Methods.

Materials
Hydrolysis of Polyester containing fabrics
Fourier Transform Infrared Spectroscopy Measurements (FTIR (Mid-IR)) and Near Infrared Measurements (NIR).
Nuclear magnetic resonance (NMR) spectroscopy.
Thermogravimetric Analysis (TGA).
Differential Scanning Calorimetry (DSC).
Optical Microscope.
Single Fiber Tensile Testing (tensile testing).
Scanning Electron Microscopy (SEM).
Pyrolysis Gas Chromatography Mass Spectrometry (Py-GC-MS).

Section 2: Supplemental Tables and Figures Descriptions

Table S1: Abbreviations used in manuscript and fabric specifications from manufacture.
Table S2: Percent yields for TPA and recovered fiber for all repeat runs. (*) notates post-consumer textile samples, and (•) notates post-consumer zipper.
Table S3: Extended values for tensile testing with estimates to identify statistical significance in relation to **Figure 6** in main text.
Table S4: Confidence Intervals for TPA yields of mixed textile materials where n=5.

Figure S1. GC-MS trace of P neat pre-consumer single fiber.

Figure S2. GC-MS trace of C neat pre-consumer single fiber.

Figure S3. GC-MS trace of 81:19 N:L neat pre-consumer fibers.

Figure S4. GC-MS trace of 88:12 P:L neat pre-consumer fibers.

Figure S5. Comparative GC-MS traces of pre-consumer fiber P (black, bottom), pre-consumer fiber C (orange, middle), and collected TPA post-reaction 25:75 P:C (blue, top), arbitrary offset applied for spectral clarity, grey dotted lines added to show similar peaks between spectra.

Figure S6. Comparative GC-MS traces of pre-consumer fiber 88:12 P:L (black, bottom) to collected TPA post-reaction (blue, top), arbitrary offset applied for spectral clarity, grey dotted lines added to show similar peaks between spectra.

Figure S7. Comparative GC-MS traces of pre-consumer fiber 81:19 N:L (black, bottom) to collected TPA 80:20 P:(N:L) post-reaction (blue, top), arbitrary offset applied for spectral clarity, grey dotted lines added to show similar peaks between spectra.

Figure S8. TGA trace of P pre-consumer material neat, heat rate 10 ˚C/min from 40 ˚C to 800 ˚C.

Figure S9. TGA trace of C pre-consumer material neat, heat rate 10 ˚C/min from 40 ˚C to 800 ˚C.

Figure S10. TGA trace of 98:2 C:L pre-consumer material neat (solid black), and 98:2 C:L pre-consumer material post-reaction (dashed blue), heat rate 10 ˚C/min from 40 ˚C to 800 ˚C.

Figure S11. DSC of P pre-consumer neat fabric, heat/cool/heat cycle, heat 20 ˚C/min, cool 10 ˚C/min, -50 ˚C to 280 ˚C.

Figure S12. DSC of (a) P:L neat pre-reaction, heat/cool/heat cycle, and (b) P:L post-reaction, heat/cool/heat cycle (L skeleton).

Figure S13. DSC of (a) N:L neat pre-reaction, heat/cool/heat cycle, and (b) N:L post-reaction, heat/cool/heat cycle.

Figure S14. DSC of (a) 98:2 C:L pre-consumer neat fabric and (b) 98:2 C:L pre-consumer fabric post-reaction, heat/cool/heat cycle, heat 20 ˚C/min, cool 10 ˚C/min, -50 ˚C to 280 ˚C.

Figure S15. DSC of (a) zipper neat pre-reaction, heat/cool/heat cycle, and (b) zipper post-reaction, heat/cool/heat cycle.

Figure S16. 88:12 P:L unreacted solid fibers post-reaction under optical microscope.

Figure S17. SEM of 88:12 P:L post-reaction collected unreacted fibers presumed to be mostly L of high yielding TPA. Top images show presumed left-over P fibers.

Figure S18. SEM collected at 1 kV of 88:12 P:L post-reaction collected unreacted fibers presumed to be mostly L of low yielding TPA. Smaller diameter fibers are presumed to be unreacted P.

Figure S19. SEM collected at 1 kV of 100 P pre-consumer before reaction (neat).

Figure S20. SEM collected at 1 kV of 100 L from Forensic Fiber Reference Collection (a) separated strands from (b) bulk.

Figure S21. SEM collected at 1 kV of 100 C pre-consumer single fiber before reaction (left) and after reaction (right).

Figure S22. SEM collected at 1 kV of 100 C post-consumer yellow shirt single fiber before reaction (left) and after reaction (right).

Figure S23. SEM collected of 88:12 P:L pre-consumer (neat) before reaction with underlying L highlighted by red circles.

Figure S24. ^{1}H NMR of deuterated dimethyl sulfoxide (DMSO-*d6* •), residual water (*w*) and artifacts (*) are also notated.

Figure S25. ^{1}H NMR of deuterated chloroform ($CDCl_3$-*d1* •) and 1,1,1,3,3,3-Hexafluoro-2-propanol (HFIP ▲), residual water (*w*) and artifacts (*) are also notated. Satellite peaks for $CDCl_3$ and HFIP can also be visualized.

Figure S26. ^{1}H NMR of zipper in deuterated chloroform ($CDCl_3$-*d1* •), with residual water (*w*) and artifacts (*) are also notated. Satellite peaks for HFIP can also be visualized.

Figure S27. ^{1}H NMR of TPA collected from reaction with 50:50 P:C pre-consumer in DMSO-*d6* (•), residual water (*w*) and artifacts resulting from DMSO-*d6* (*) are also notated.

Figure S28. ^{1}H NMR of TPA collected from reaction of 100 P post-consumer zipper in DMSO-*d6* (•), residual water (*w*) and artifacts resulting from DMSO-*d6* (*) are also notated.

Figure S29. ^{1}H NMR of TPA collected from reaction of 52:48 P:C post-consumer grey shirt in DMSO-*d6* (•), residual water (*w*) and artifacts resulting from DMSO-*d6* (*) are also notated.

Figure S30. ^{1}H NMR of TPA collected from reaction of 100 P post-consumer black and red pants in DMSO-*d6* (•), residual water (*w*) and artifacts resulting from DMSO-*d6* (*) are also notated.

Figure S31. ^{1}H NMR of TPA from zipper in DMSO-*d6* (•), residual water (*w*) and artifacts resulting from DMSO-*d6* (*) are also notated.

Figure S32. ATR-FTIR (mid-IR) spectra of 52:48 P:C post-consumer material before reaction (black, bottom), and 52:48 P:C post-consumer material after reaction (blue, top). Distinct peaks associated with C boxed in orange (left) and peak associate with P boxed in green (right), absorbance spectra is arbitrarily offset for clarity.

Figure S33. ATR-FTIR (mid-IR) spectra of zipper from post-consumer backpack before experiment (black, bottom), and zipper from after experiment (blue, top), absorbance spectra is arbitrarily offset for clarity.

Figure S34. ATR-FTIR (mid-IR) spectra of TPA from zipper (black, bottom), and TPA from after experiment on zipper (blue, top), absorbance spectra is arbitrarily offset for clarity.

Figure S35. ATR-FTIR (mid-IR) spectra of 92:8 C:L pre-consumer fabric before reaction (black, bottom), and 92:8 C:L pre-consumer fabric after experiment (blue, top),), absorbance spectra is arbitrarily offset for clarity.

Figure S36. NIR spectra of 52:48 P:C grey post-consumer shirt (black, bottom), and 52:48 P:C grey post-consumer shirt post-reaction (blue, top). Distinct peaks associated with C boxed in orange (left) and peak associate with P boxed in green (right), absorbance spectra is arbitrarily offset for clarity.

Figure S37. NIR spectra of post-consumer zipper from backpack before (black, bottom), and post-consumer zipper from backpack post-reaction (blue, top), absorbance spectra is arbitrarily offset for clarity.

Figure S38. NIR spectra of 92:8 C:L pre-consumer material before reaction (black, bottom), and 92:8 C:L pre-consumer material post-reaction (blue, top), absorbance spectra is arbitrarily offset for clarity.

Figure S39. Characterization of TPA produced from pre-consumer materials containing N and L. (a) MID-IR spectra and (b) TGA with TPA from distributer (black), TPA from 88:12 P:L (blue), and TPA from a 80:20 P:(N:L) (orange). Discussed alongside **Figure 8.**

Figure S40. Characterization of post-consumer material's post-reaction. (a) MID-IR spectra and (b) TGA with TPA from distributer (black), TPA from 100 P:C pants (blue), and TPA from 52:48 t-shirt (orange, dashed). Discussed alongside **Figure 9.**

Figure S41. Characterization of post-consumer cotton shirt pre- (black) and cotton shirt post-reaction (blue), (a) NIR, (b) MID-IR spectra (full spectra inlet), and (c) TGA.

Section 3: Data Availability

Section 1: Materials and Methods.

Disclaimer. Certain commercial equipment, instruments, or materials are identified in this paper to specify the experimental procedure adequately. Such identification is not intended to imply recommendation or endorsement by the National Institute of Standards & Technology, nor is it intended to imply that the materials or equipment identified are necessarily the best available for the purpose.

Materials. All chemicals were purchased from Sigma-Aldrich and used as received without further purification unless otherwise noted. Pre-consumer fabrics of known provenance were purchased from Testfabrics, Inc. (West Pittston, PA). Fabric specifications can be found in supplemental information (Table S1). Laboratory-made blends were prepared by weight in either a shredded or pre-cut format before placing in the reaction vessel. These fibers being shredded rather than interwoven should not change the results of hydrolysis. Material blends ratios will be listed as X:Y which correlate to the material type that followed Xx:Yy. Example: 50:50 P:C is a 50 % by 50 % by mass mixture of polyester and cotton. There are also pre-blended fiber constructs

from Testfabrics and will have the same nomenclature. Such as, material type 88:12 P:L has the elastane component incorporated into the construction of the polyester fabric. This material type is always prepared in a pre-cut format. Additional textiles such as 92:8 C:L were also investigated. Post-consumer textiles were purchased from a local thrift store. A presumed, and verified in the discussion, polyester-based zipper was also studied. A known provenance pure elastane was used from the Forensic Fiber Reference Collection.[1]

Hydrolysis of polyester containing fabrics. Various components were mixed into nominally 25 mL of deionized water (DI H_2O) in a glass tube. This included about 2 wt % of laboratory-made blends (e.g., P:C, P:(N:L)), made using shredded or pre-cut squares, (500 mg). Pre-consumer fabric sheets such as P:L and C:L are also used in this work. Lab-made blends are prepared by weighing of each component to make a desired blend by mass. For example, for a 50:50 P:C blend about 250 mg of shredded or pre-cut squares of P are mixed with about 250 mg of shredded or pre-cut squared of C and placed into the reaction vessel. Then, 10 wt. % of KOH (2.77g) which makes a 2 mol/L base solution, and a 0.2 wt. % of BTBAC (41.5 mg) which makes 0.005 mol/L solution are added.[18] BTBAC acts as a phase-transfer catalyst (PTC) which is utilized to facilitate reactions between reactants that are in different, immiscible phases, as in this case between the basic solution and solid fiber. The reaction vessel was placed in an oil bath at about 90 ˚C for around 4 h and quenched by placing in ice water. The tube was capped loosely with a septa cap, the pH and volume of the reaction were checked periodically and did not change. The solution was filtered through a glass microfiber filter (2.7 µm pore size) and any solid unreacted material was further processed. The resulting filtrate was acidified with H_2SO_4 to a pH of around 3 to precipitate the TPA. The resulting TPA precipitate was filtered through a glass microfiber filter, rinsed 3 times with 20 mL of DI H_2O, and dried in a vacuum oven at around 70 ˚C for 18 h before weighing. Each experiment was repeated a minimum of 5 times per material type (Table S2). It is important to note that while a PTC was used herein, it is not necessary for this process to work, one would just need longer reaction times. Others have shown the difference in reaction times and lower temperatures when using a PTC.[28–32] The reaction was performed for 100:0 P:C

under the same reaction conditions without PTC present to investigate the role of the PTC, and the yield of TPA was reduced to only 56.2 %. The post-consumer zipper, was cut into about 1.22 cm to 2.54 cm length strips to fit within the reaction vessel solution and weighed about 500 mg. All subsequent steps were the same.

Fourier Transform Infrared Spectroscopy Measurements (FTIR (Mid-IR)) and Near Infrared Measurements (NIR). Analysis of fabrics by FTIR using a built-in attenuated total reflectance (ATR) accessory was performed on a Nicolet iS50 series spectrometer utilizing OMNIC standard software of mid-IR region with a KBr beamsplitter. Standard uncertainties associated with this measurement are ± 1 % in peak intensity and ± 4 cm^{-1} in wavenumber. The instrument software's ATR baseline correction was applied to all spectra. The benchtop near infrared (NIR) attachment was utilized with the same spectrometer with integrating sphere, an indium gallium arsenide (InGaAs) detector, and a calcium difluoride (CaF_2) beamsplitter. Each material was tested with 128 scans at 4.00 cm^{-1} resolution.

Nuclear magnetic resonance (NMR) spectroscopy. Spectra were recorded on a 600 MHz Bruker spectrometer. Chemical shifts were referenced to the residual solvent signal of $CDCl_3$ (about 7.26 ppm), and DMSO-*d6* (nominally 2.50 ppm), as indicated in the spectra captions.

Thermogravimetric Analysis (TGA). A 550 series thermogravimetric analyzer, Waters, with a heat ramp setting, from the room temperature, of 10 ˚C/min to 700 ˚C or 800 ˚C were performed under N_2 environment for TPA specimens and under compressed air for fiber materials. Silica wafers were used atop the fibers (pre-tared with TGA pan) to prevent movement of fibers during the experiment. The standard uncertainty associated with this measurement is ± 0.01 % in mass and ± 1 ˚C in temperature.

Differential Scanning Calorimetry (DSC). DSC was carried out using a TA Instruments Discovery 2500 DSC. Samples were prepared in aluminum hermetic pans from TA Instruments. Typical test conditions involved a heat-cool-heat-cool-heat procedure of with a heating rate setting of 20 ˚C/min and a cooling rate of 10 ˚C/min. For example, from 40 ˚C to 280 ˚C, then 280 ˚C to

-50 ˚C, then from -50 ˚C to 280 ˚C for multiple cycles. For temperature measurements a standard uncertainty of ± 0.1 ˚C is assumed.

Optical Microscope. A Leica DM4000 M optical microscope with InFocus software was used with a 10× objective. Samples were sandwiched between two microscope slides for analysis. No support oils were used.

Single Fiber Tensile Testing (tensile testing). Single fiber specimens were separated from the pre- and post-reaction specimen threads. Damage from handling was minimized by carefully removing the twist in the thread prior to extraction of the single fibers. Specimens were tested using a displacement driven load frame specifically designed for testing single fibers. The load frame has a 2.1 N load cell with a resolution of 1 μN, and the crosshead displacement measurement has a resolution of 0.1 μm. The testing procedure was compliant with ASTM D3822 using a constant crosshead displacement of about 3 mm/min and a gauge length of approximately 7 mm. The single fibers were gripped directly, without tabs, using 5 mm polymethyl methacrylate pneumatic grips. Only failure force was measured in this study, so individual fiber diameter was not measured for each specimen due to fibers having similar fiber size distributions determined by scanning electron microscopy.

Scanning Electron Microscopy (SEM). Samples were affixed to the stubs using carbon adhesive and imaged using a JEOL 7800F with the Everhart-Thornley detector. The electron acceleration voltage was 1 kV or 2 kV depending on sample morphology for charge compensation. These images were processed using Image J software.

Pyrolysis Gas Chromatography Mass Spectrometry (Py-GC-MS). Primary textile and alkaline hydrolysis byproduct samples were chemically analyzed by pyrolysis (CDS 6150 Pyroprobe, CDS Analytical LLC, Oxford, PA, USA) gas chromatography-mass spectrometry (Thermo Trace 1310 gas chromatograph / TSQ 8000evo triple quadrupole mass spectrometer, Thermo Fisher Scientific, Waltham, MA USA). Py-GC-MS breaks down materials from the pyrolytic nature of the instrument, therefore, background peaks will be visible. Solid samples or

suspensions in water were placed into quartz tubes and loaded into the pyrolyzer autosampler. Single-step analyses consisted of a rapid heating ramp (40 ˚C/min) to 700 ˚C and held at this temperature for about 30 s. A nominally 5% diphenyl dimethyl polysiloxane phase column (30 m x 0.25 mm ID x 0.25 μm film: RXI-5 15HT), 33:1 split ratio, and helium carrier gas were employed with a 15 ˚C/min oven ramp up to 290 ˚C and held for about 10 min for chromatographic separation. Traditional 70 eV EI mass spectra were collected and matched against the NIST mass spectral library for compound identification. The pyrolysis chamber was cleaned following each analysis at around 1000 ˚C for about 30 s and blank quartz tube controls were run between samples.

Section 2: Supplemental Tables and Figures Descriptions.

Table S1: Abbreviations used in manuscript and fabric specification from manufacture.

Material Abbreviation	Material Description and/or Specification
P	Polyester
C	Cotton
N	Nylon
L	Elastane
BTBAC	Benzyltributylammonium chloride
TPA	Terephthalic Acid
PET	Polyethylene terephalate
BHET	Bishydroxyethyl terephthalate
SI	Supporting information
P	Poly Georgette (TESTFABRICS 1411003)
C	Bleached Combed Cotton Fabric (TESTFABRICS 1403022)
P:L	Polyester/LYCRA (aka elastane) (88%/ 12%) with optical brightener (TESTFABRICS 700-5018)
C:L	Cotton/LYCRA (aka elastane) (92%/ 8%) Leotard Fabric
N:L	Nylon/LYCRA (aka elastane (81%/ 19%) Knit Bathing Suit Fabric (TESTFABRICS 1415021)

Table S2: Percent yields for TPA and recovered fiber for all repeat runs. (*) notates post-consumer textile samples, and (•) notates post-consumer zipper.

Material	Blend Ratio	TPA Yield (%)	Fiber Yield (%)
P	100	98.2	-
		96.8	
		96.4	
		96.2	
		97.2	
$P_{pants\ post\text{-}consumer}$	100	92.2	-
		92.8	
		94.7	
		90.1	
		93.2	
$P_{zipper\ post\text{-}consumer}$	100	17.8	-
		11.6	
		10.4	
		16.2	
		14.9	
P:C	75:25	94.8	93.9
		95.2	94.7
		96.9	95.2
		96.8	95.0
		93.9	100.9
P:C	50:50	96.5	99.3
		94.5	92.9
		96.3	94.7
		92.6	92.8
		95.4	96.0
P:C	25:75	93.4	97.8
		90.5	95.2
		94.4	95.6
		95.0	94.1
		89.2	100.1
$P{:}C_{shirt\ post\text{-}consumer}$	52:48	88.1	80.8
		84.8	84.6
		85.2	87.5
		86.1	88.1
		87.4	84.5
P:(N:L)	80:20	96.5	98.5
		95.9	98.2
		96.9	97.8
		95.8	99.3
		96.9	100.8
P:(N:L)	50:50	94.3	99.0
		95.5	98.3
		96.2	98.6
		95.0	98.5

		95.0	98.1
P:(N:L)	25:75	90.1	99.0
		90.7	98.9
		91.0	98.9
		92.8	98.3
		94.1	99.6
P:L	88:12	86.9	90.1
		88.7	92.5
		88.4	97.7
		85.8	91.2
		89.8	92.7

Table S3: Extended values for tensile testing with estimates to identify statistical significance in relation to **Figure 6** in main text.

Material	Average Force (N)	Weibull Scale Parameter (N)			Weibull Shape Parameter		
		Estimate	CI		Estimate	CI	
Neat Cotton	0.0457	0.051	0.046	0.057	2.81	2.28	3.47
Cotton Post-Reaction (0:100 P:C)	0.0372	0.042	0.038	0.046	3.09	2.50	3.83
Cotton Post-Reaction (25:75 P:C)	0.0383	0.043	0.039	0.046	3.63	2.91	4.52

Table S4. Confidence Intervals for TPA yields of mixed textile materials where n=5.

Material Blended Ratio	Mean Yield (%)	Confidence Interval (95 %)		Standard Deviation
		Lower Bound (%)	Upper Bound (%)	
P 100	97.0	96.0	97.8	0.709
$P_{pants\ post\text{-}consumer}$ 100	92.6	90.7	94.5	1.49
$P_{zipper\ post\text{-}consumer}$ 100	14.2	10.7	17.6	2.78
P:C 75:25	95.5	94.1	97.0	1.17
P:C 50:50	95.1	93.3	96.8	1.42
P:C 25:75	92.5	89.7	95.3	2.26
$P{:}C_{shirt\ post\text{-}consumer}$ 52:48	86.3	84.8	87.9	1.26
P:(N:L) 80:20	96.4	95.8	97.0	0.473
P:(N:L) 50:50	95.2	94.4	96.0	0.629
P:(N:L) 25:75	91.7	89.9	93.6	1.48
P:L 88:12	87.9	86.2	89.7	1.41

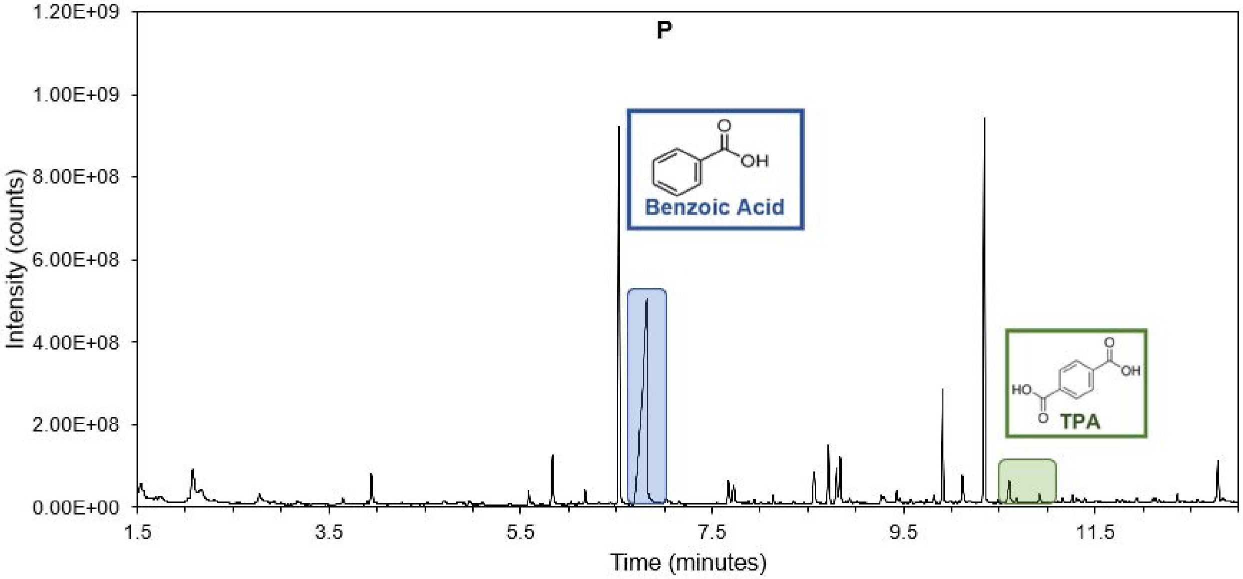

Figure S1. GC-MS trace of P neat pre-consumer single fiber.

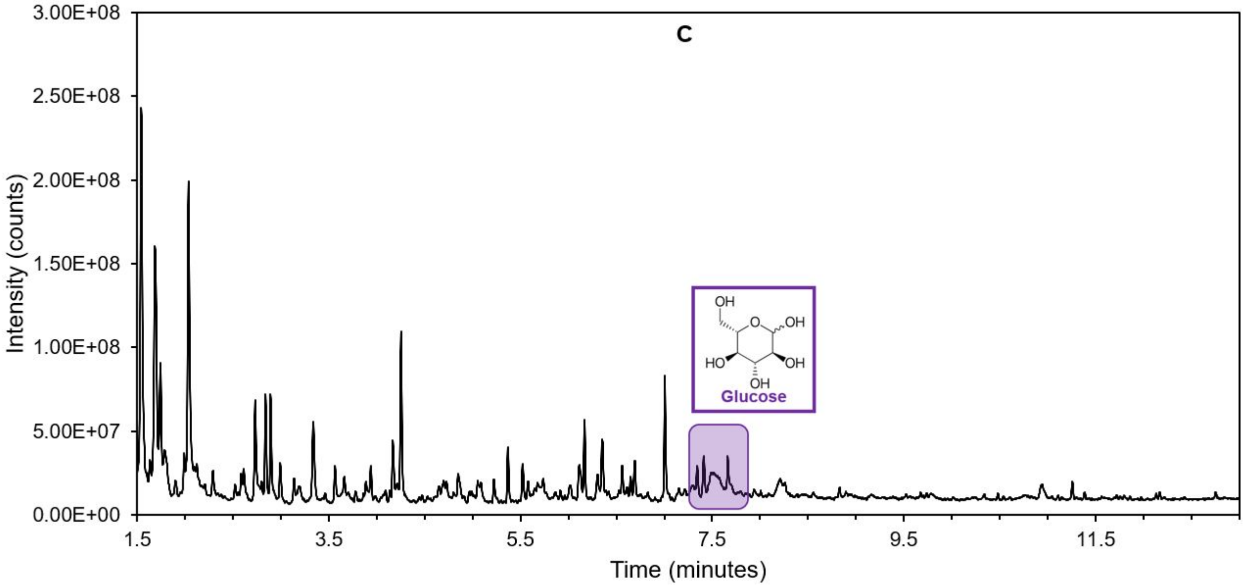

Figure S2. GC-MS trace of C neat pre-consumer single fiber.

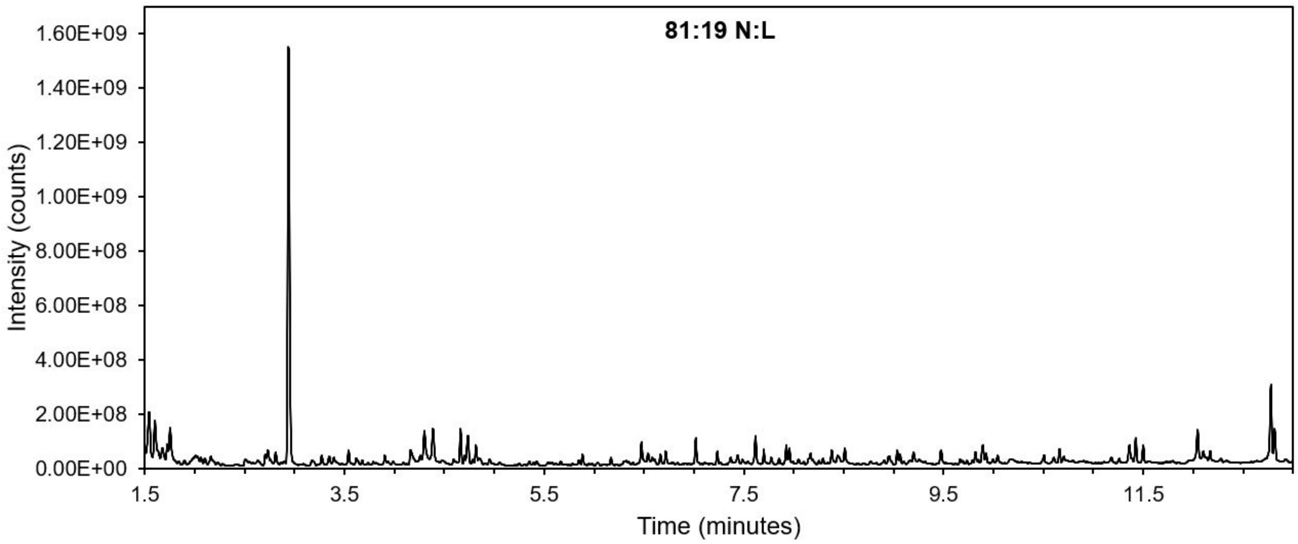

Figure S3. GC-MS trace of 81:19 N:L neat pre-consumer fibers.

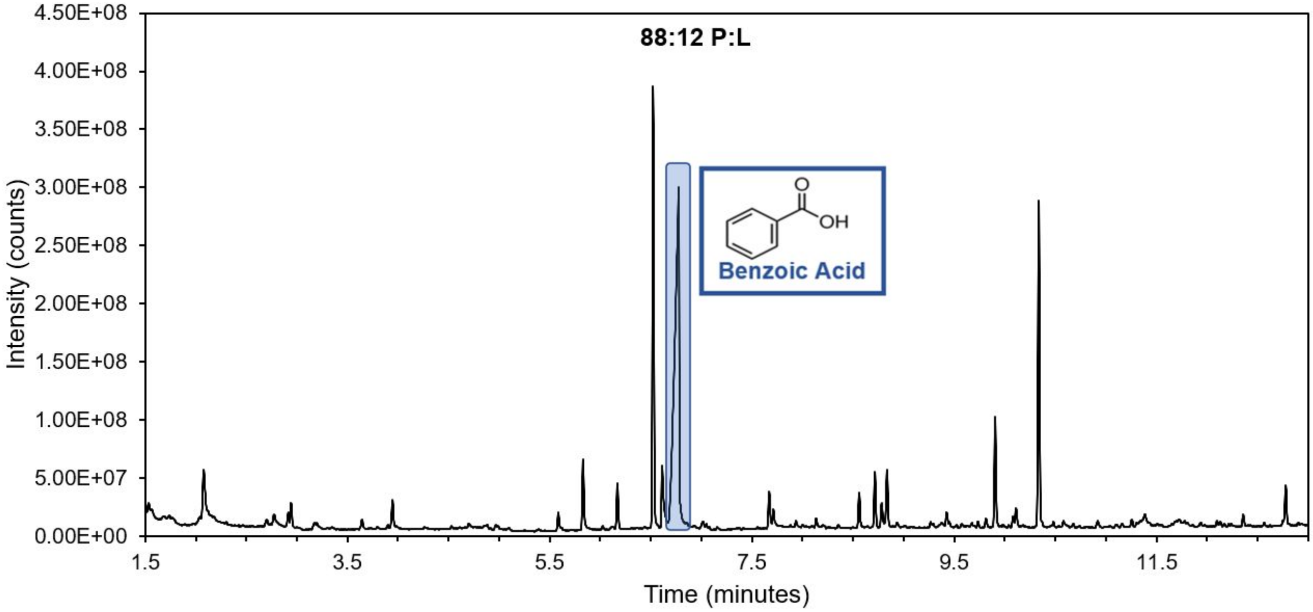

Figure S4. GC-MS trace of 88:12 P:L neat pre-consumer fibers.

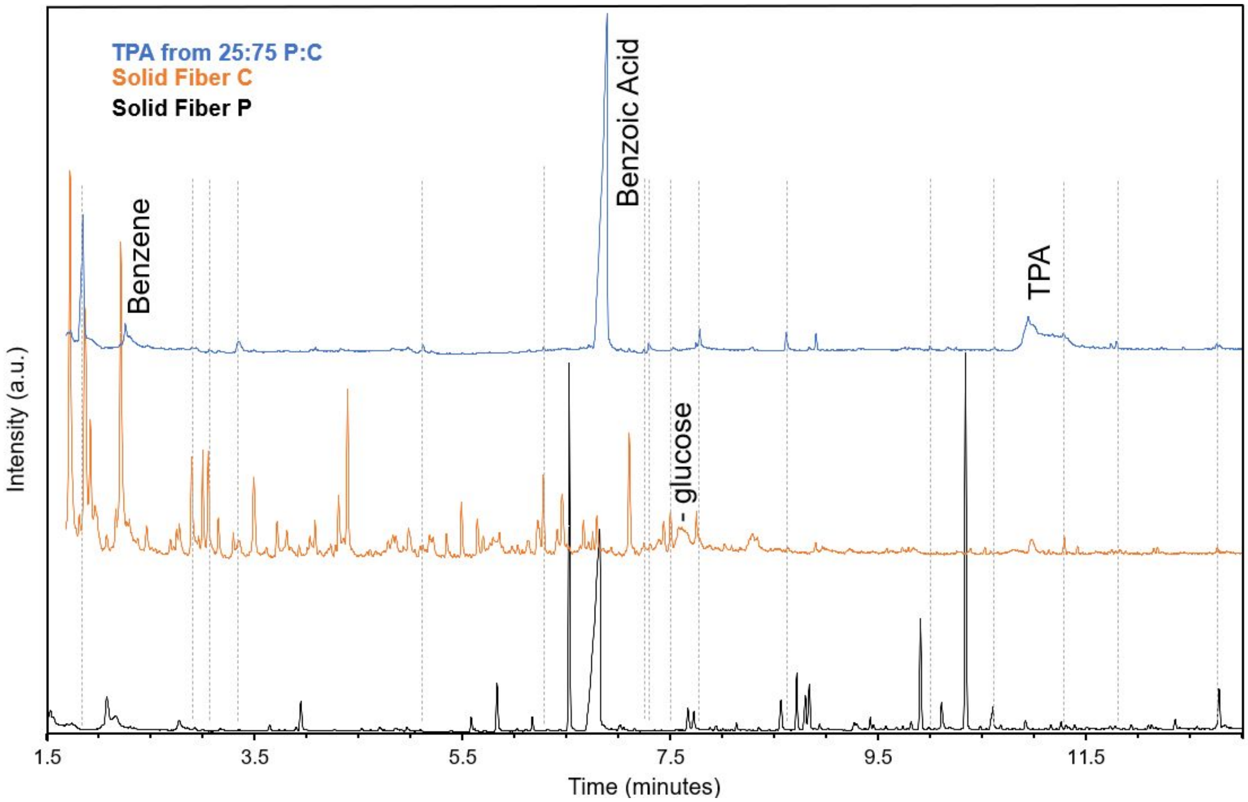

Figure S5. Comparative GC-MS traces of pre-consumer fiber P (black, bottom), pre-consumer fiber C (orange, middle), and collected TPA post-reaction 25:75 P:C (blue, top), arbitrary offset applied for spectral clarity, grey dotted lines added to show similar peaks between spectra.

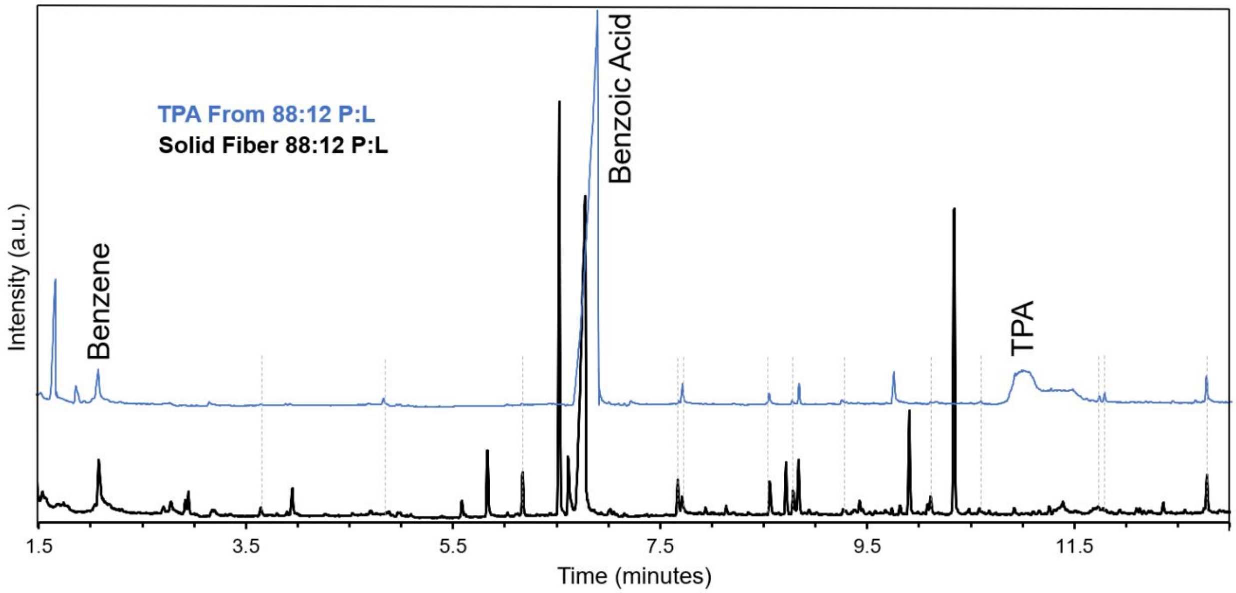

Figure S6. Comparative GC-MS traces of pre-consumer fiber 88:12 P:L (black, bottom) to collected TPA post-reaction (blue, top), arbitrary offset applied for spectral clarity, grey dotted lines added to show similar peaks between spectra.

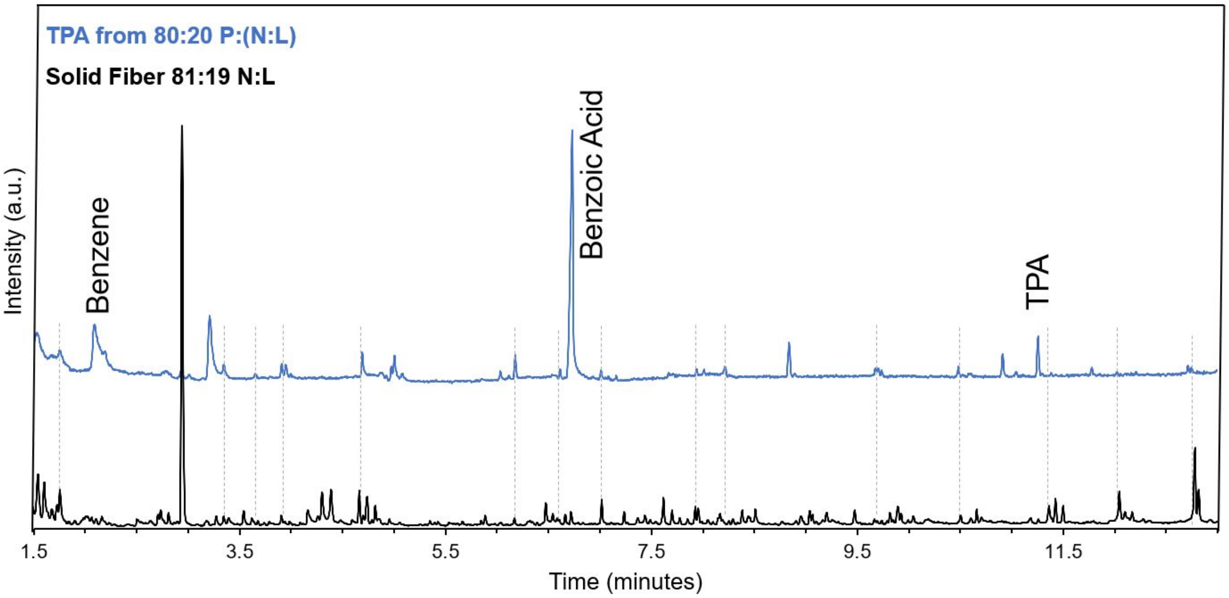

Figure S7. Comparative GC-MS traces of pre-consumer fiber 81:19 N:L (black, bottom) to collected TPA 80:20 P:(N:L) post-reaction (blue, top), arbitrary offset applied for spectral clarity, grey dotted lines added to show similar peaks between spectra.

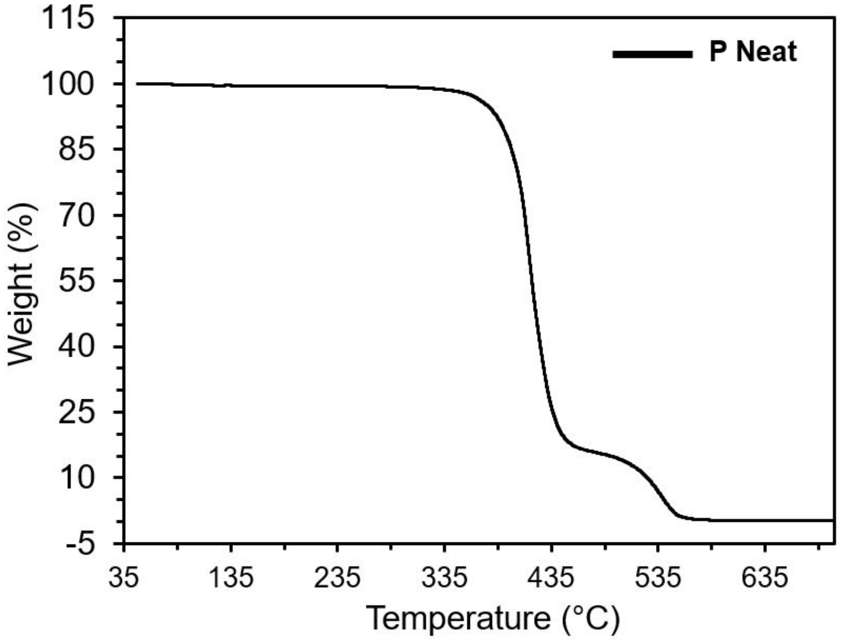

Figure S8. TGA trace of P pre-consumer material neat, heating rate 10 ˚C/min from 40 ˚C to 800 ˚C.

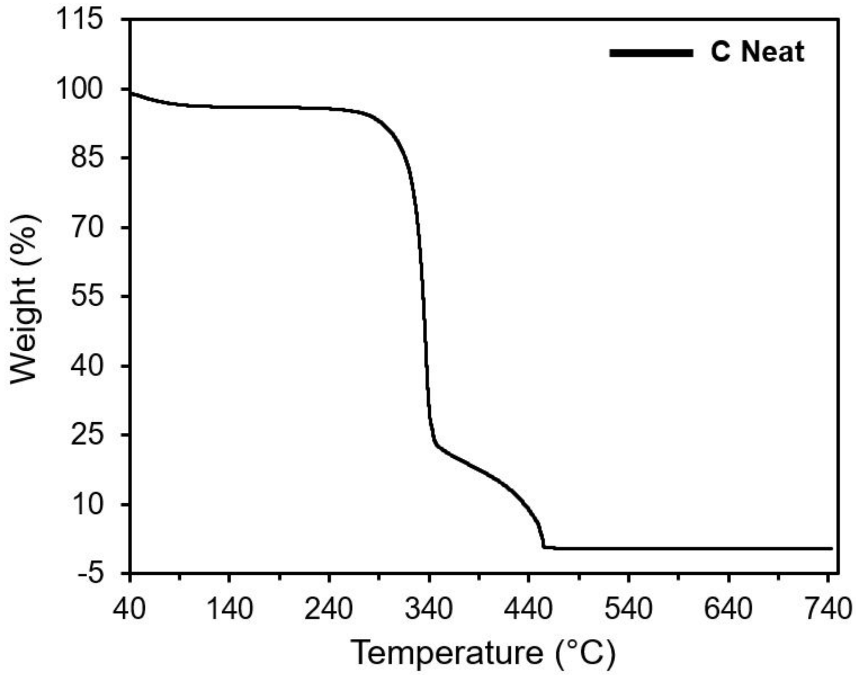

Figure S9. TGA trace of C pre-consumer material neat, heating rate 10 ˚C/min from 40 ˚C to 800 ˚C.

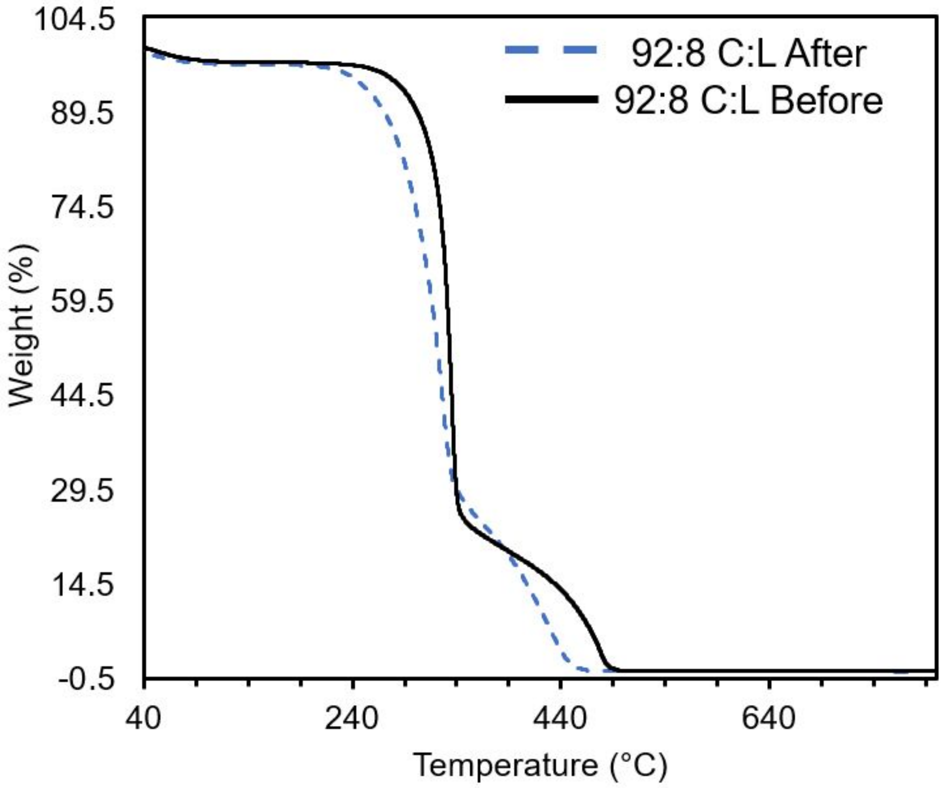

Figure S10. TGA trace of 98:2 C:L pre-consumer material neat (solid black), and 98:2 C:L pre-consumer material post-reaction (dashed blue), heating rate 10 ˚C/min from 40 ˚C to 800 ˚C.

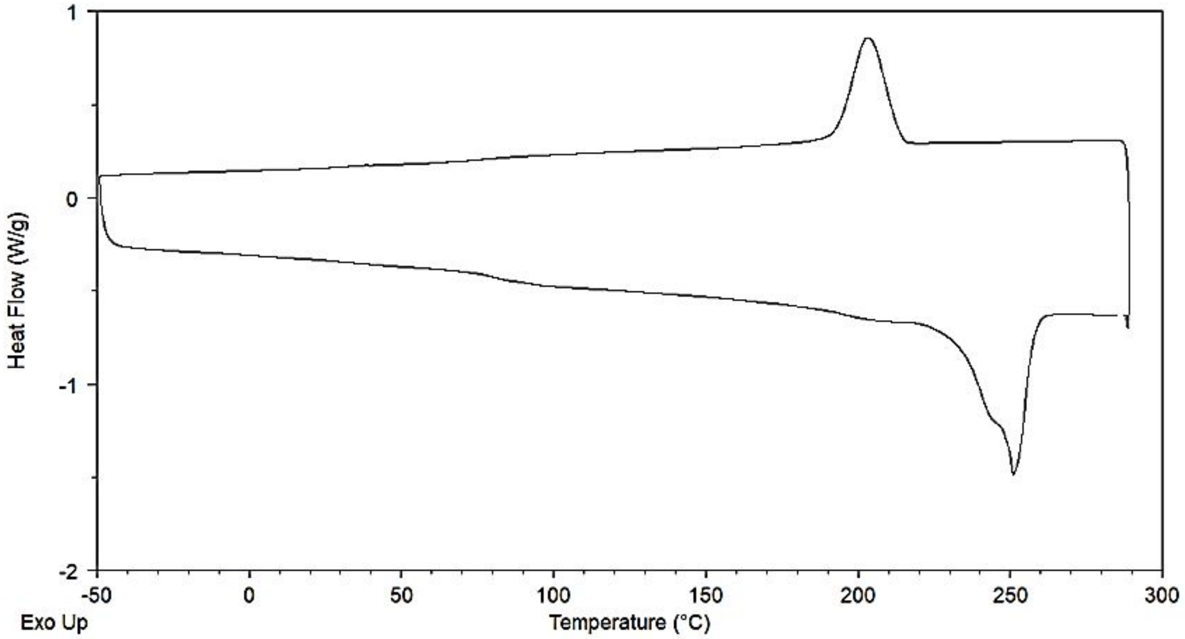

Figure S11. DSC of P pre-consumer neat fabric, final heat/cool cycle, heat 20 ˚C/min, cool 10 ˚C/min, -50 ˚C to 280 ˚C.

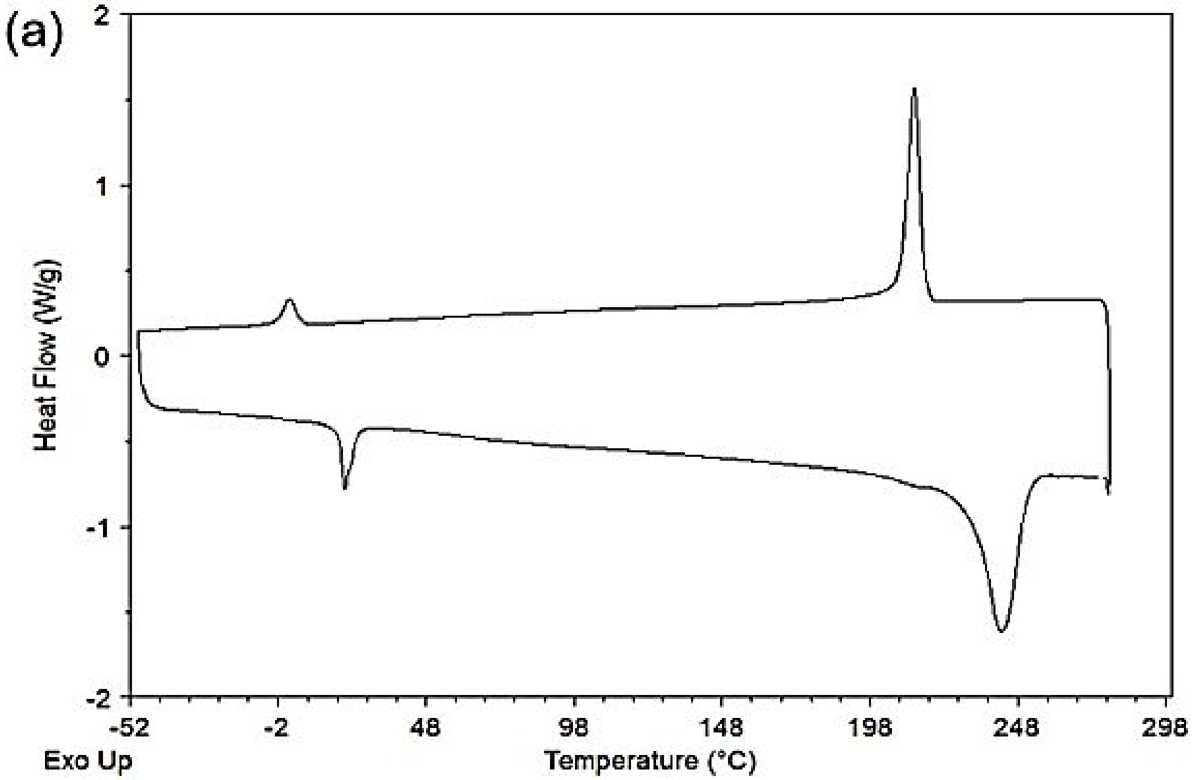

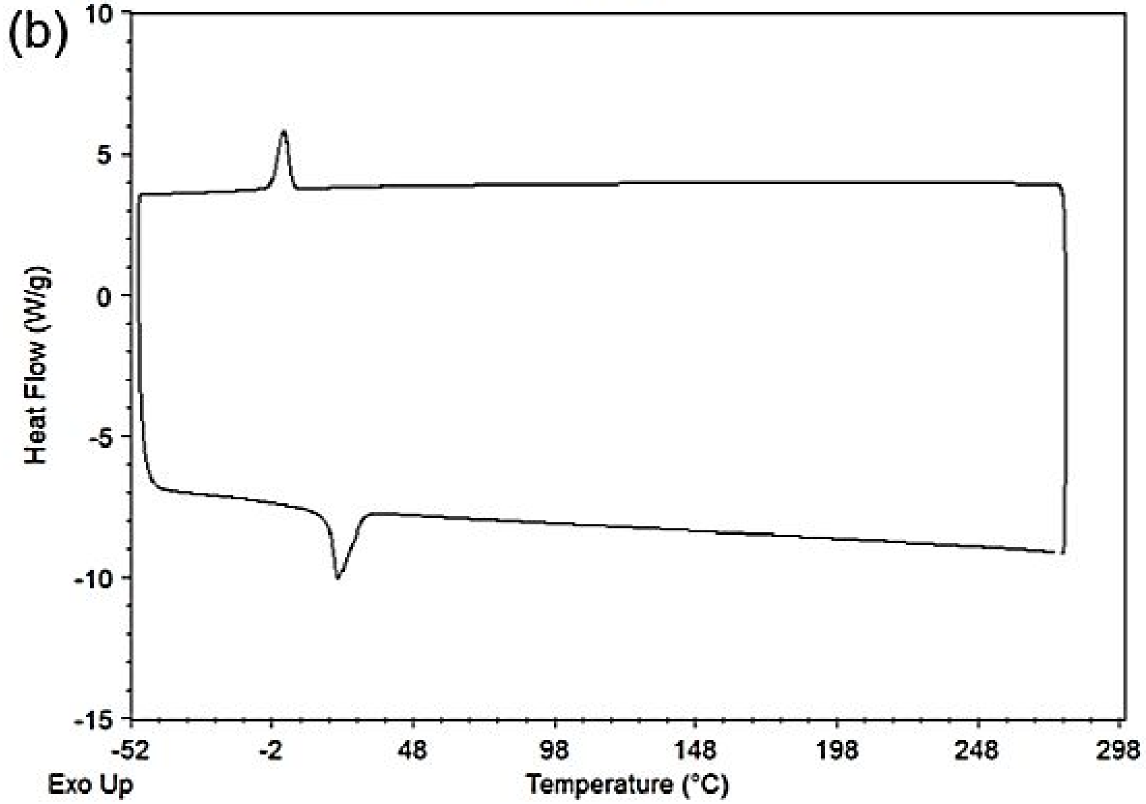

Figure S12. DSC of (a) P:L neat pre-reaction, final heat/cool cycle, and (b) P:L post-reaction, heat/cool/heat cycle (L skeleton).

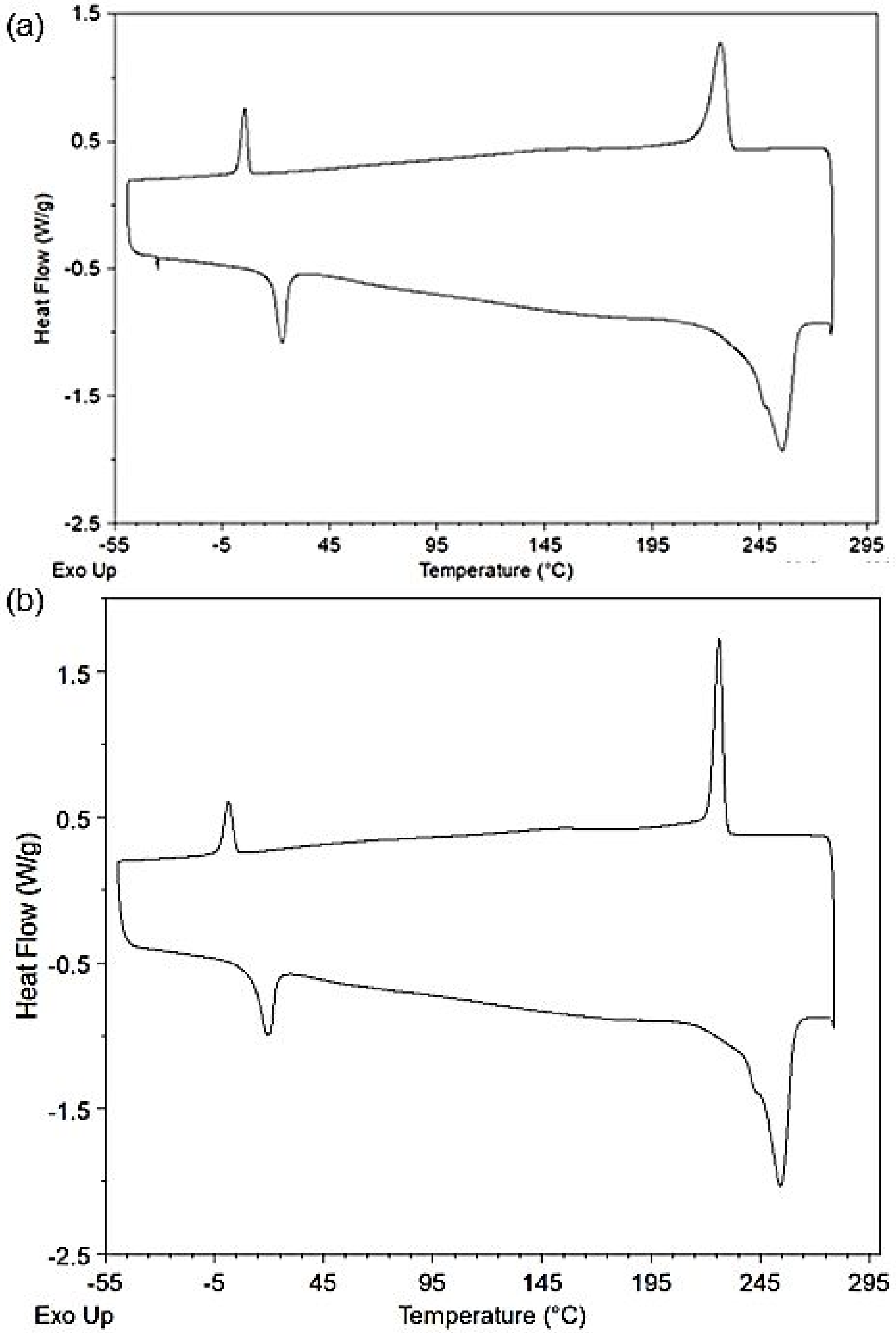

Figure S13. DSC of (a) N:L neat pre-reaction, final heat/cool cycle, and (b) N:L post-reaction, heat/cool/heat cycle.

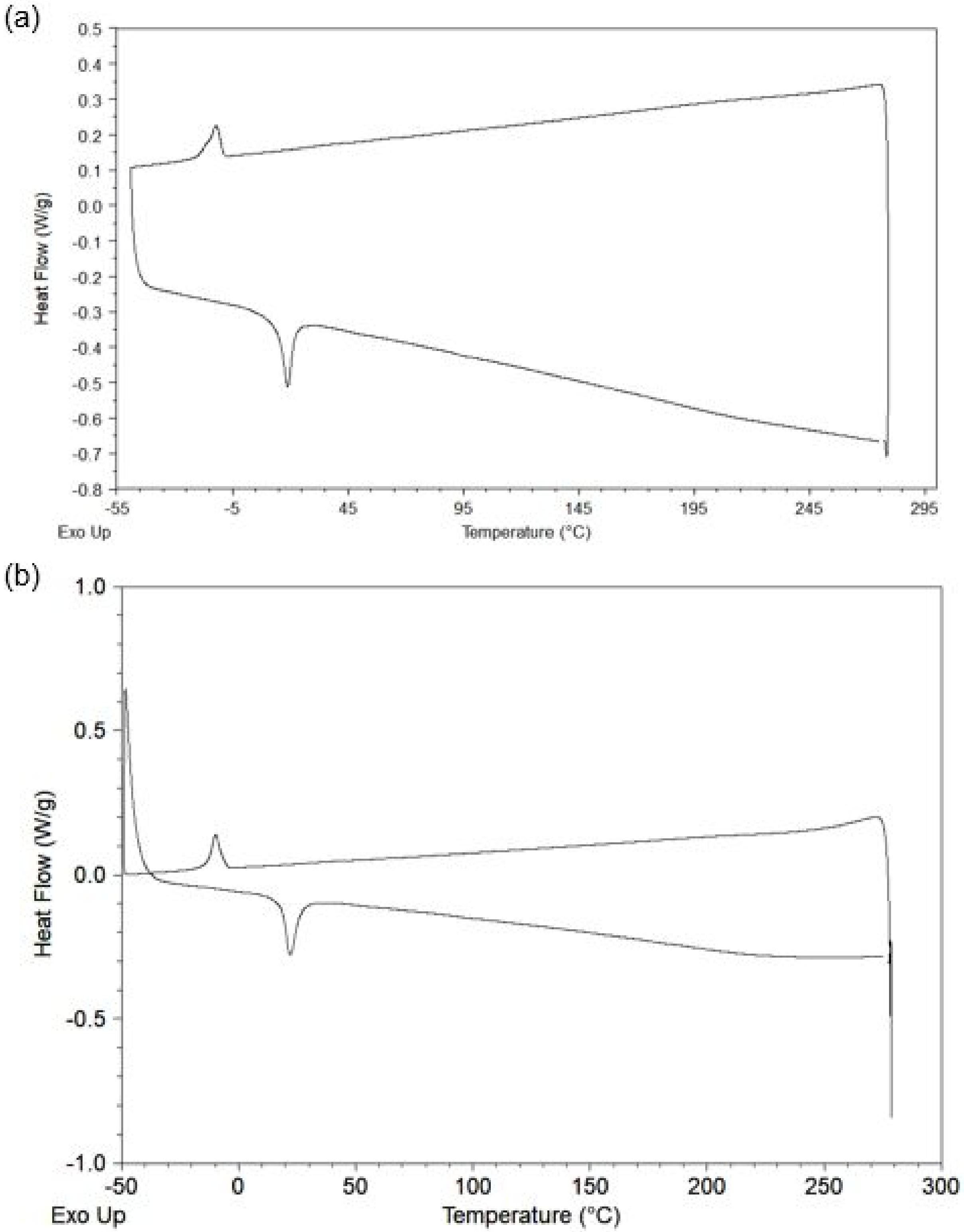

Figure S14. DSC of (a) 98:2 C:L pre-consumer neat fabric and (b) 98:2 C:L pre-consumer fabric post-reaction, final heat/cool cycle, heat 20 ˚C/min, cool 10 ˚C/min, -50 ˚C to 280 ˚C.

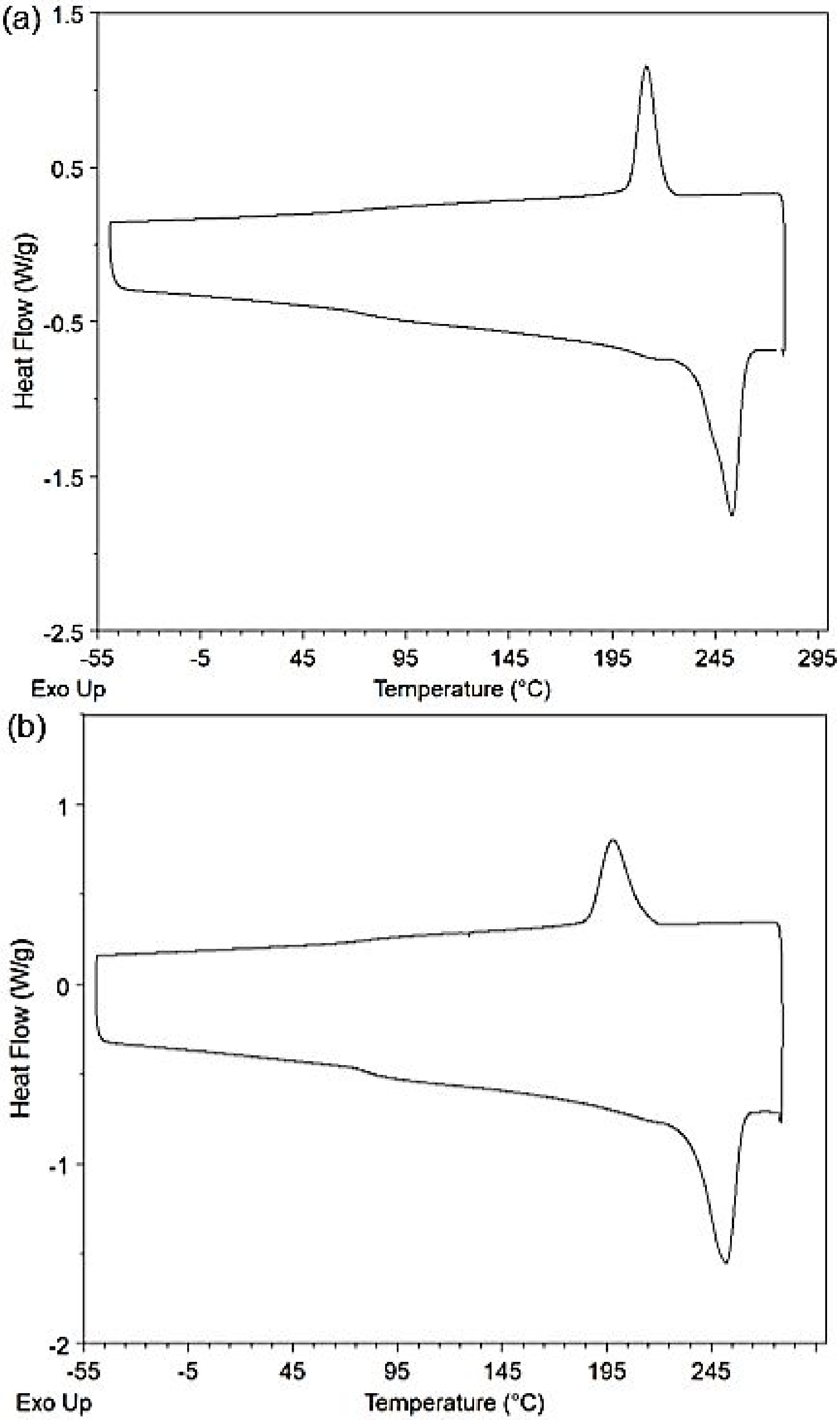

Figure S15. DSC of (a) zipper neat pre-reaction, final heat/cool cycle, and (b) zipper post-reaction, heat/cool/heat cycle.

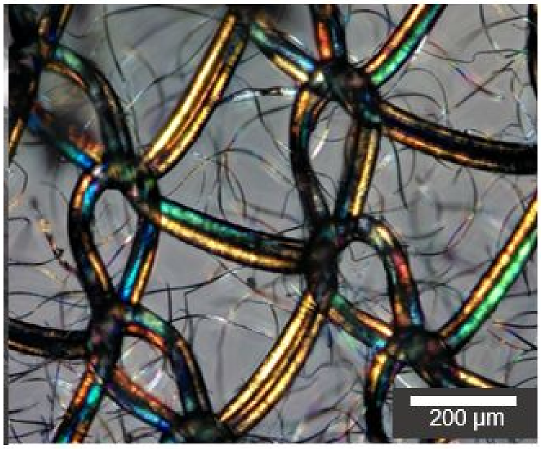

Figure S16. 88:12 P:L unreacted solid fibers post-reaction under optical microscope.

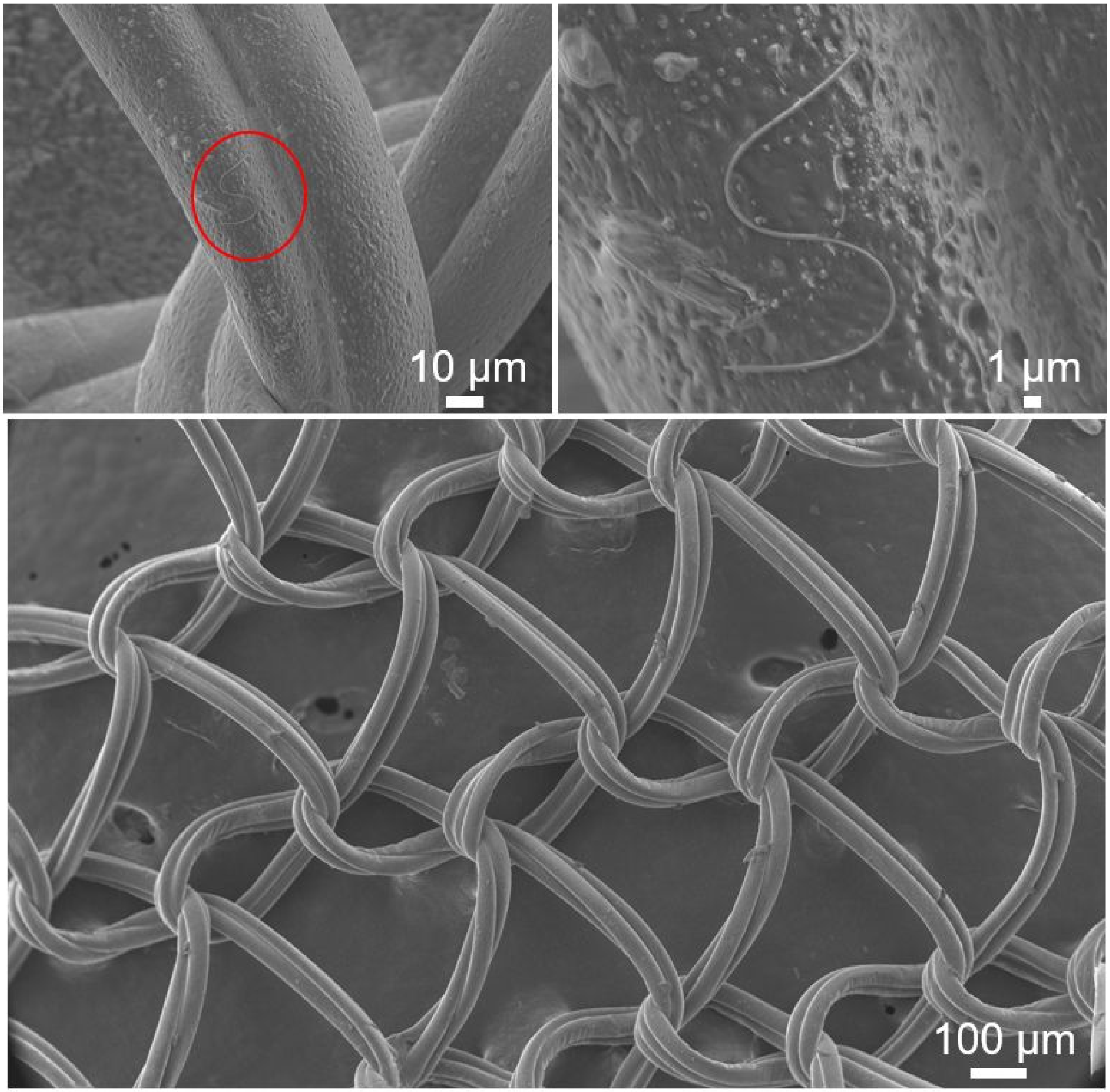

Figure S17. SEM of 88:12 P:L post-reaction collected unreacted fibers presumed to be mostly L of high yielding TPA. Top images show presumed left-over P fibers.

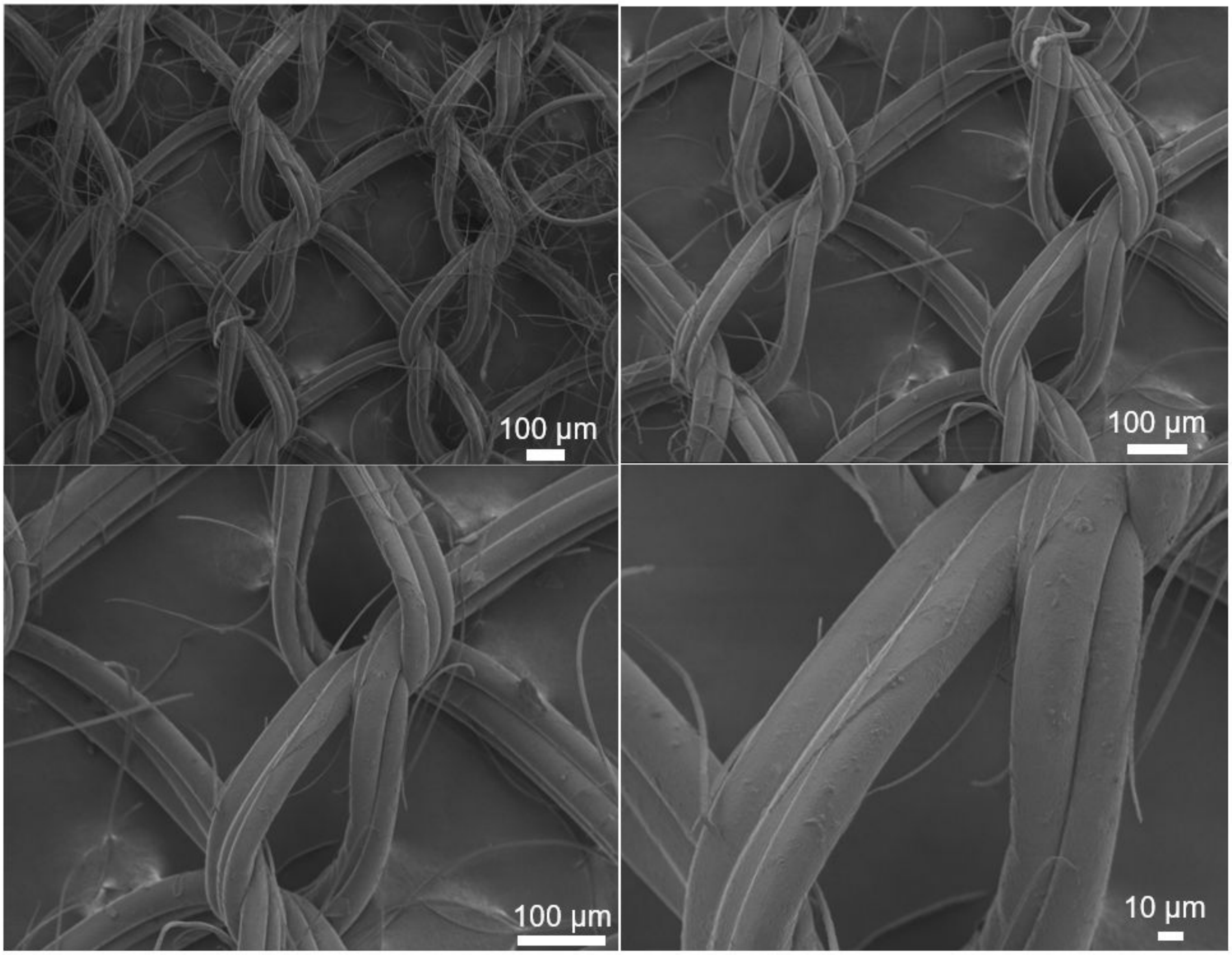

Figure S18. SEM collected at 1 kV of 88:12 P:L post-reaction collected unreacted fibers presumed to be mostly L of low yielding TPA. Smaller diameter fibers are presumed to be unreacted P.

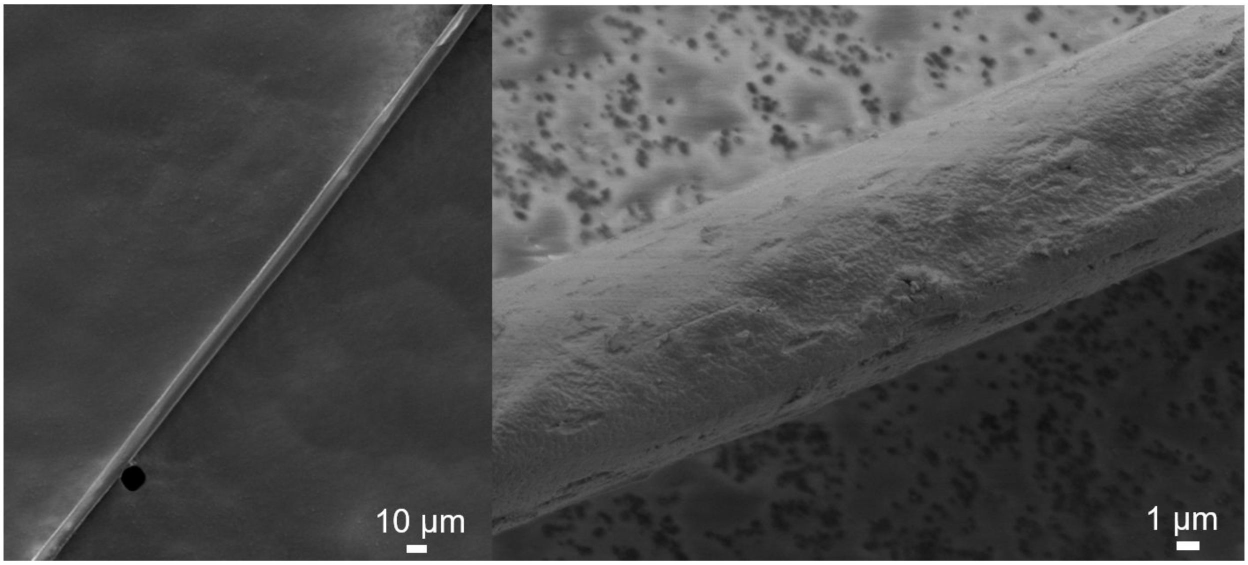

Figure S19. SEM collected at 1 kV of 100 P pre-consumer before reaction (neat).

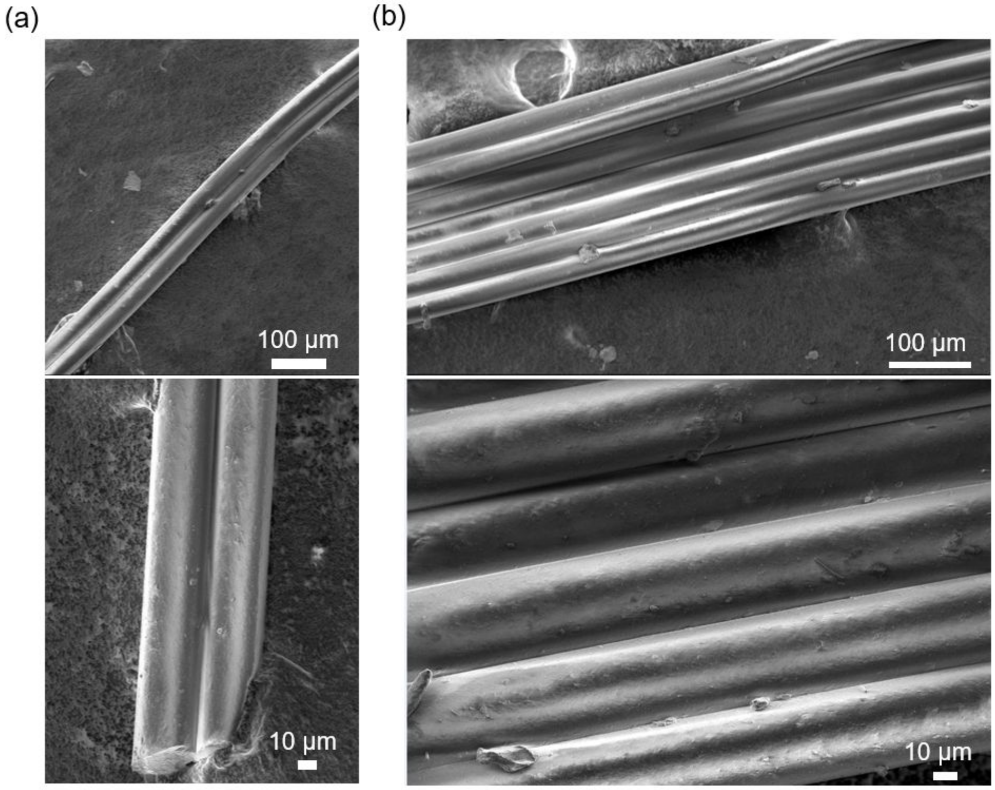

Figure S20. SEM collected at 1 kV of 100 L from Forensic Fiber Reference Collection (a) separated strands from (b) bulk.

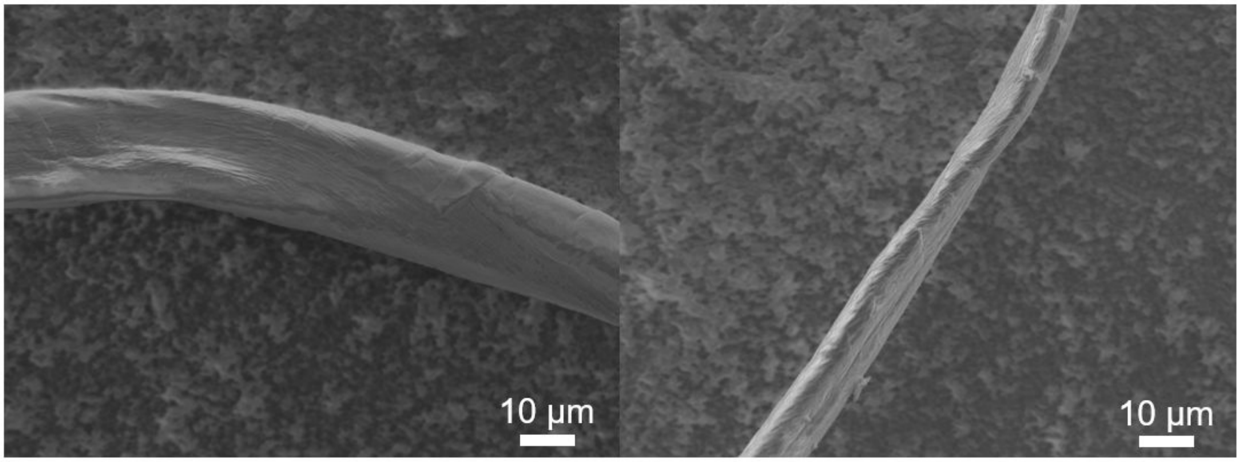

Figure S21. SEM collected at 1 kV of 100 C pre-consumer single fiber before reaction (left) and after reaction (right).

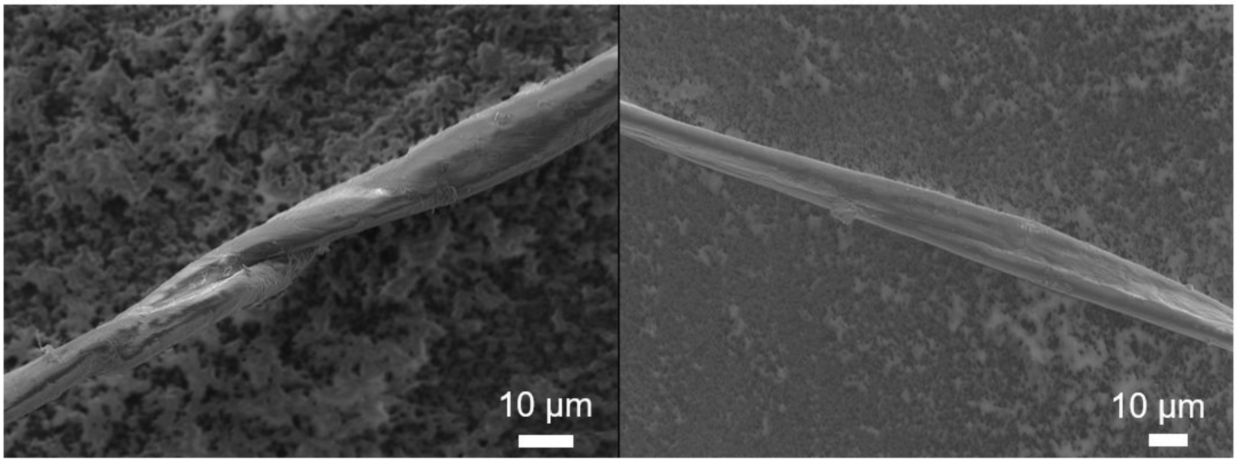

Figure S22. SEM collected at 1 kV of 100 C post-consumer yellow shirt single fiber before reaction (left) and after reaction (right).

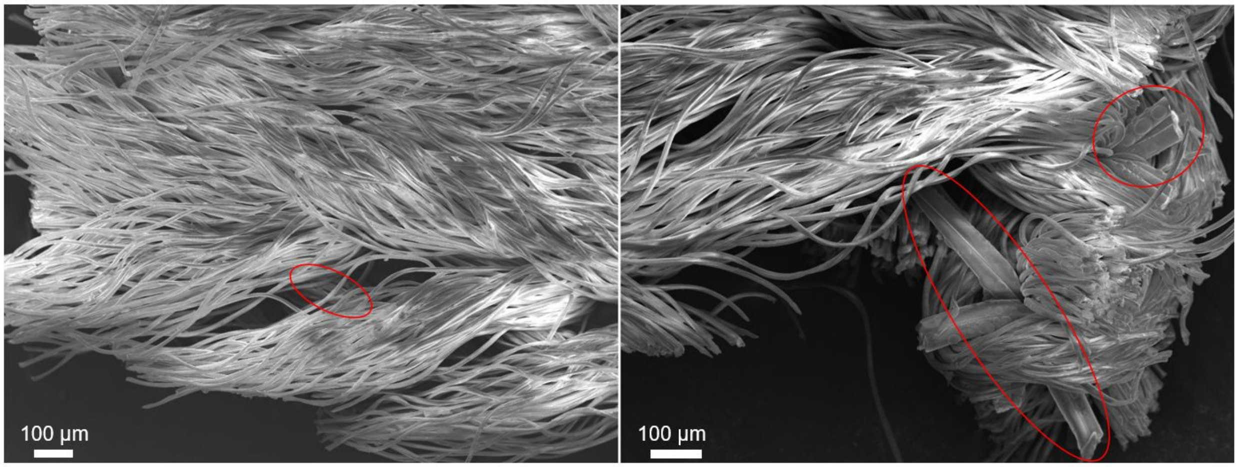

Figure S23. SEM collected of 88:12 P:L pre-consumer (neat) before reaction with underlying L highlighted by red circles.

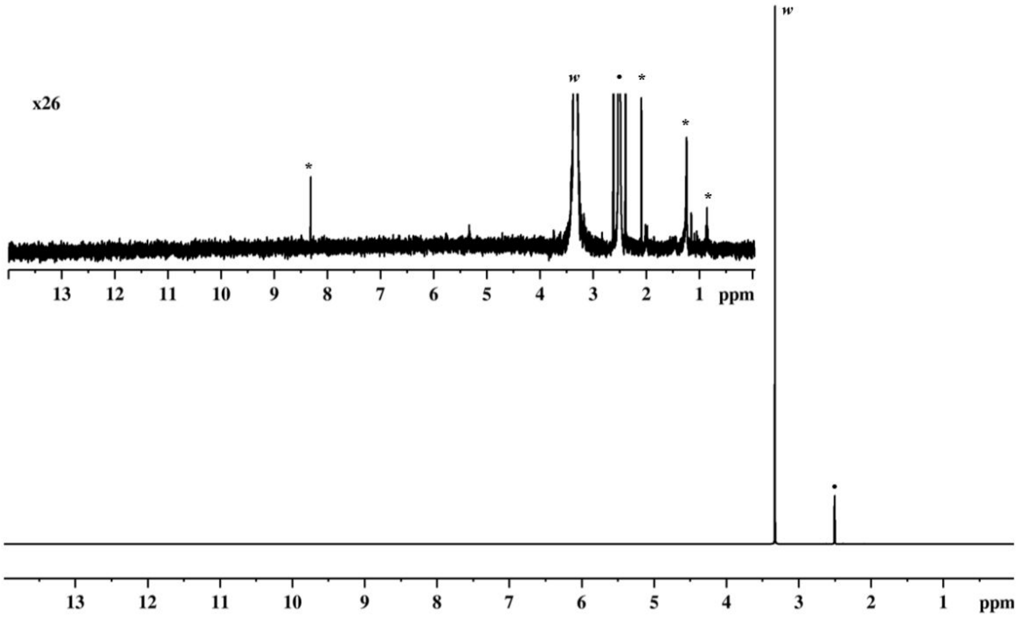

Figure S24. ^1H NMR of deuterated dimethyl sulfoxide (DMSO-*d6* •), residual water (*w*) and artifacts (*) are also notated.

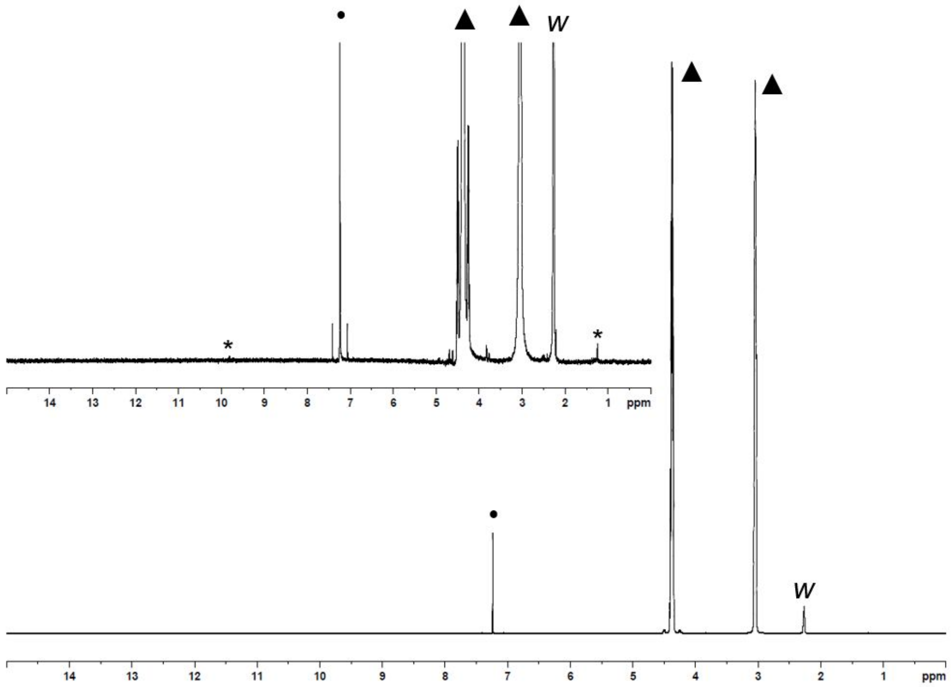

Figure S25. ^{1}H NMR of deuterated chloroform ($CDCl_3$-*d1* •) and 1,1,1,3,3,3-Hexafluoro-2-propanol (HFIP ▲), residual water (*w*) and artifacts (*) are also notated. Satellite peaks for $CDCl_3$ and HFIP can also be visualized.

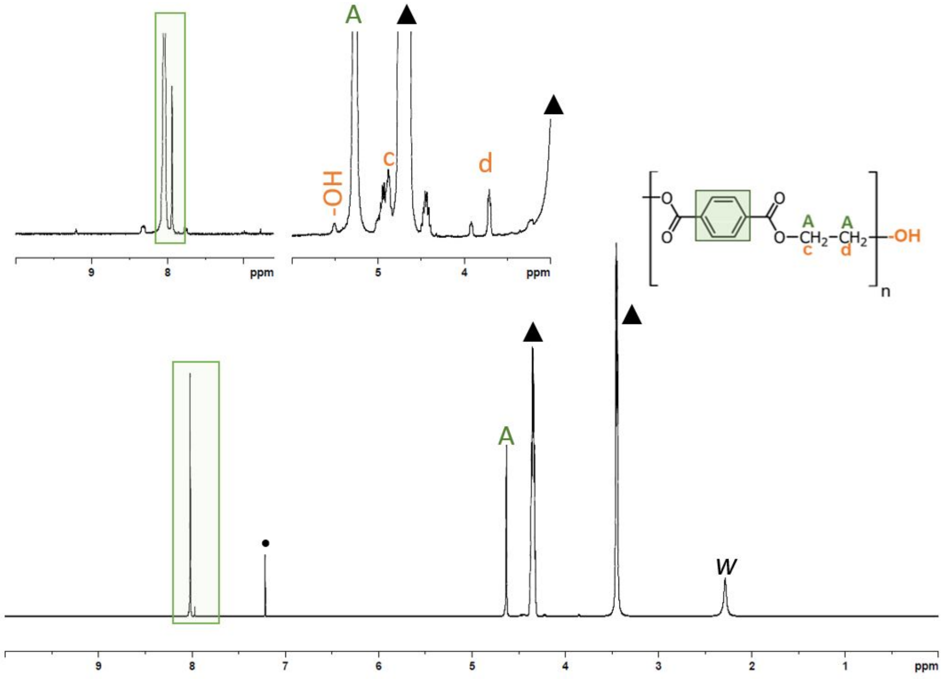

Figure S26. ^{1}H NMR of zipper in deuterated chloroform ($CDCl_3$-*d1* •), with residual water (*w*) and artifacts (*) are also notated. Satellite peaks for HFIP can also be visualized.

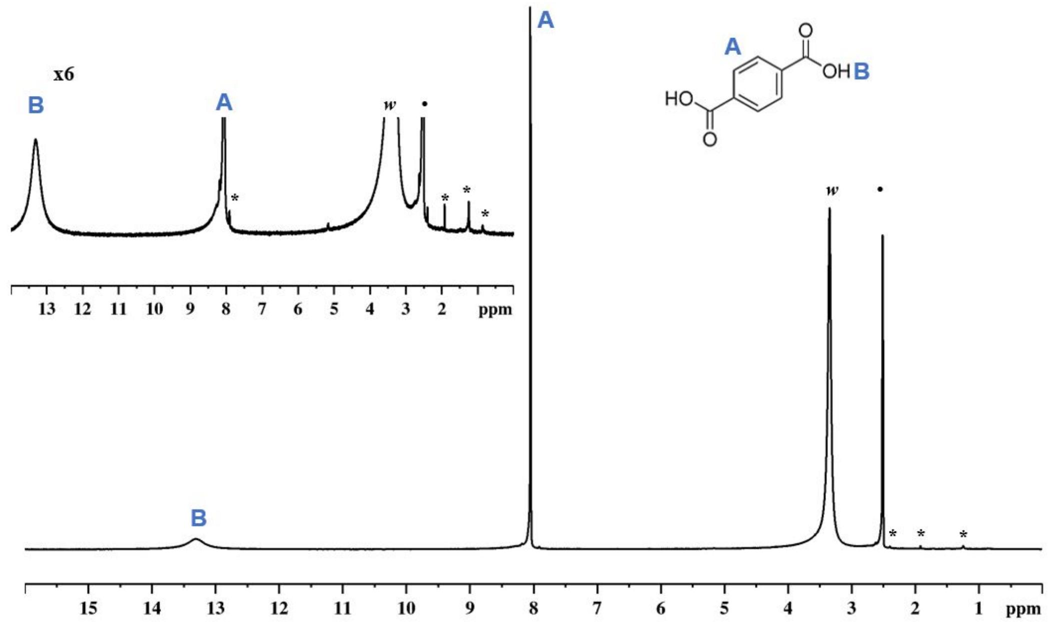

Figure S27. ^{1}H NMR of TPA collected from reaction with 50:50 P:C pre-consumer in DMSO-*d6* (•), residual water (*w*) and artifacts resulting from DMSO-*d6* (*) are also notated.

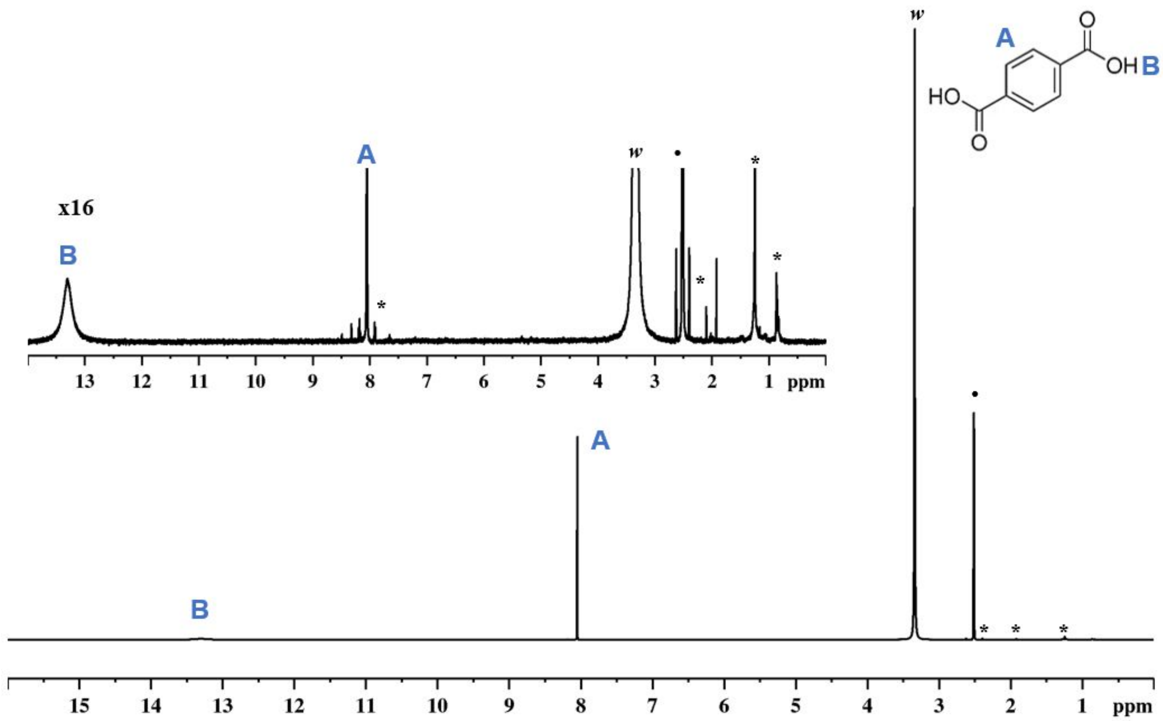

Figure S28. ^{1}H NMR of TPA collected from reaction of 100 P post-consumer zipper in DMSO-*d6* (•), residual water (*w*) and artifacts resulting from DMSO-*d6* (*) are also notated.

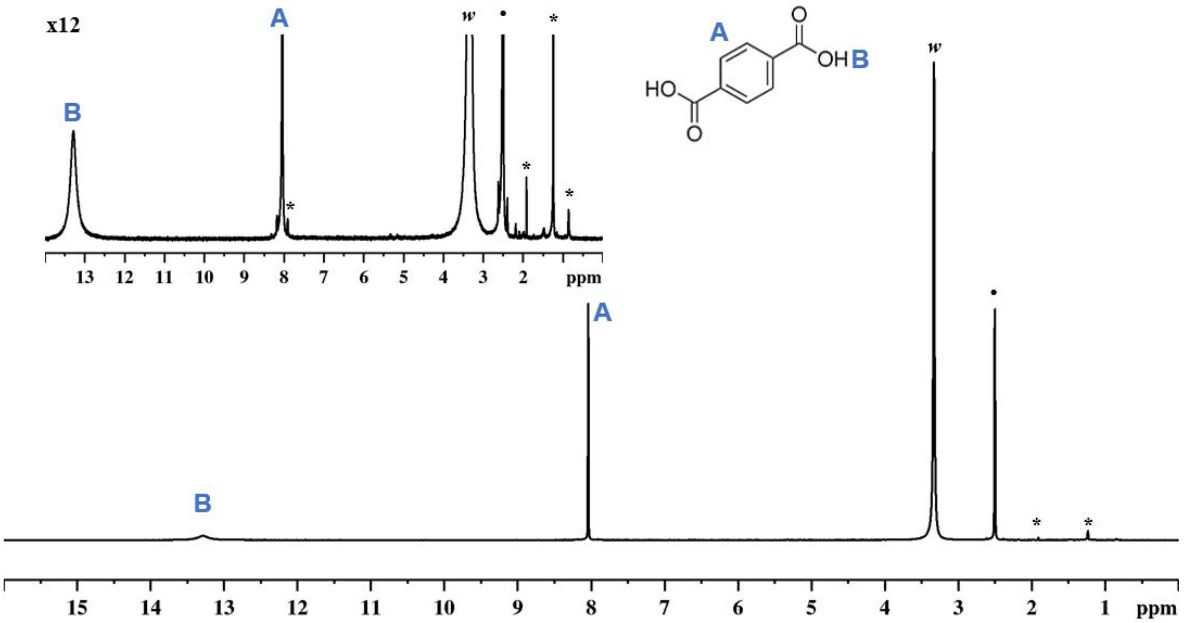

Figure S29. ^{1}H NMR of TPA collected from reaction of 52:48 P:C post-consumer grey shirt in DMSO-*d6* (•), residual water (*w*) and artifacts resulting from DMSO-*d6* (*) are also notated.

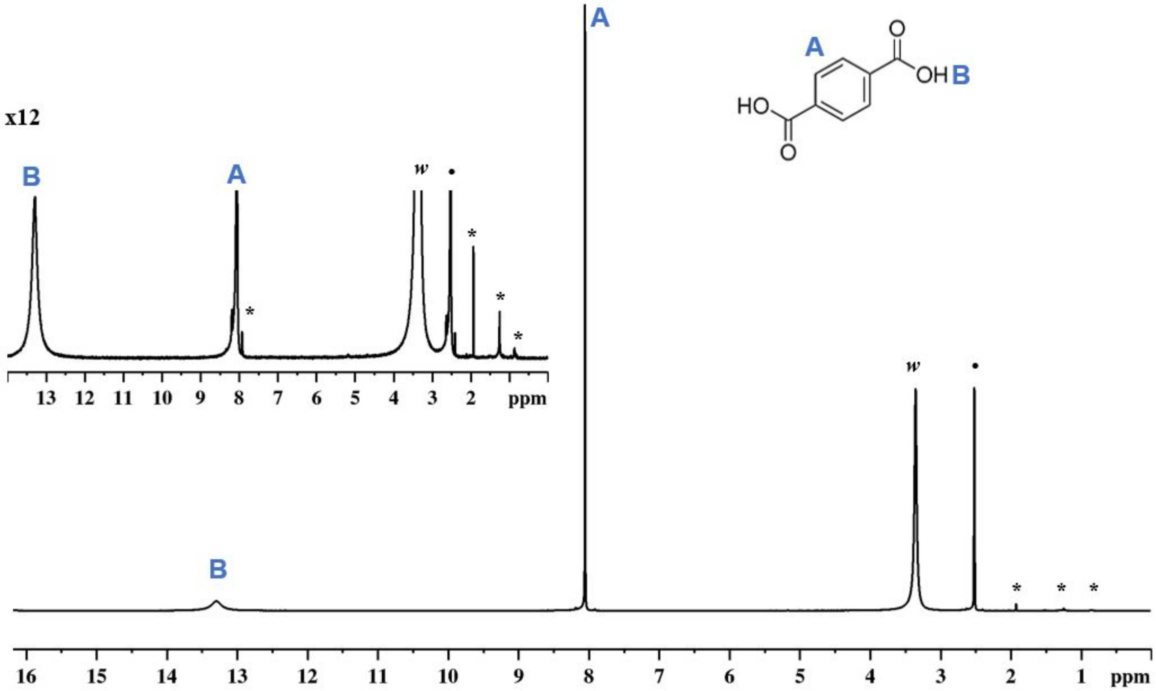

Figure S30. ^{1}H NMR of TPA collected from reaction of 100 P post-consumer black and red pants in DMSO-*d6* (•), residual water (*w*) and artifacts resulting from DMSO-*d6* (*) are also notated.

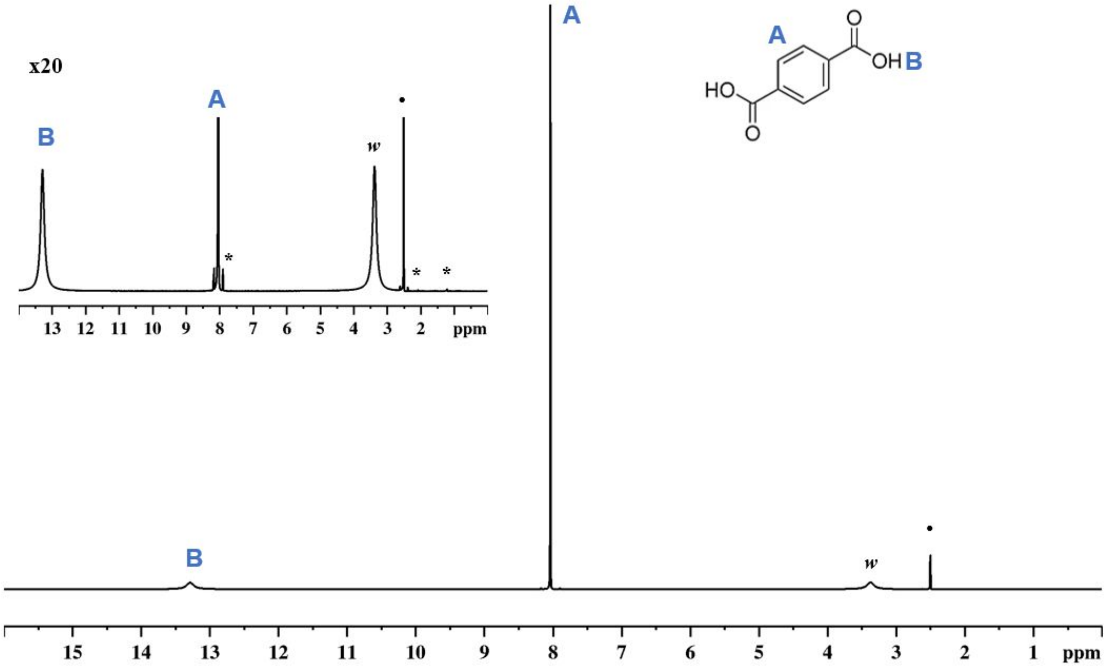

Figure S31. ^{1}H NMR of TPA from zipper in DMSO-*d6* (•), residual water (*w*) and artifacts resulting from DMSO-*d6* (*) are also notated.

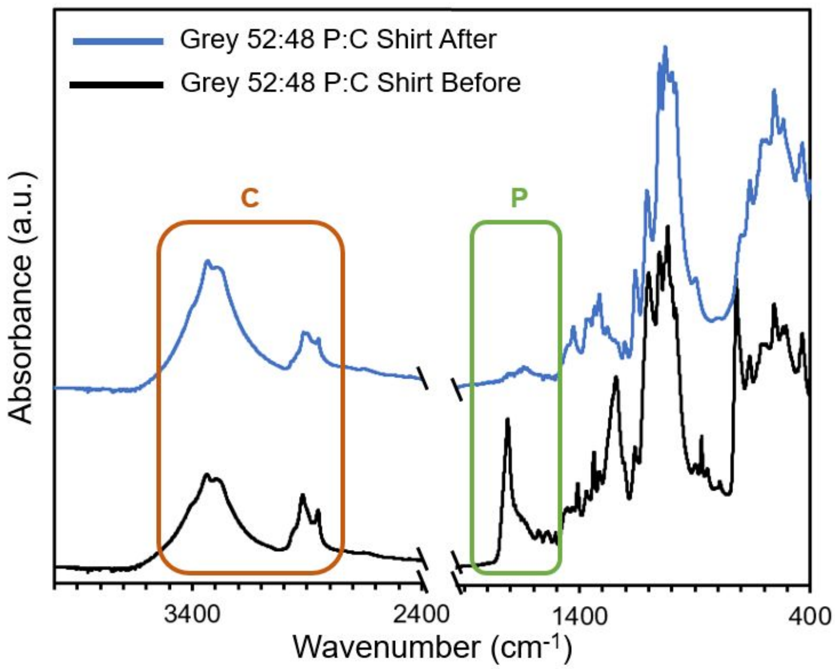

Figure S32. ATR-FTIR (mid-IR) spectra of 52:48 P:C post-consumer material before reaction (black, bottom), and 52:48 P:C post-consumer material after reaction (blue, top). Distinct peaks associated with C boxed in orange (left) and peak associate with P boxed in green (right), absorbance spectra are arbitrarily offset for clarity.

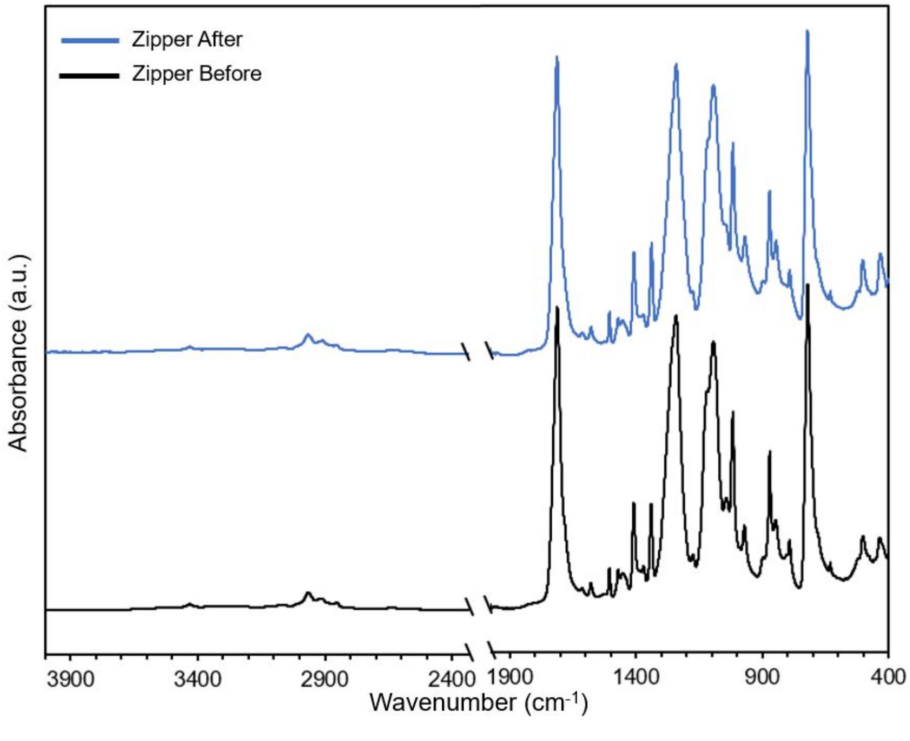

Figure S33. ATR-FTIR (mid-IR) spectra of zipper from post-consumer backpack before experiment (black, bottom), and zipper from after experiment (blue, top), absorbance spectra are arbitrarily offset for clarity.

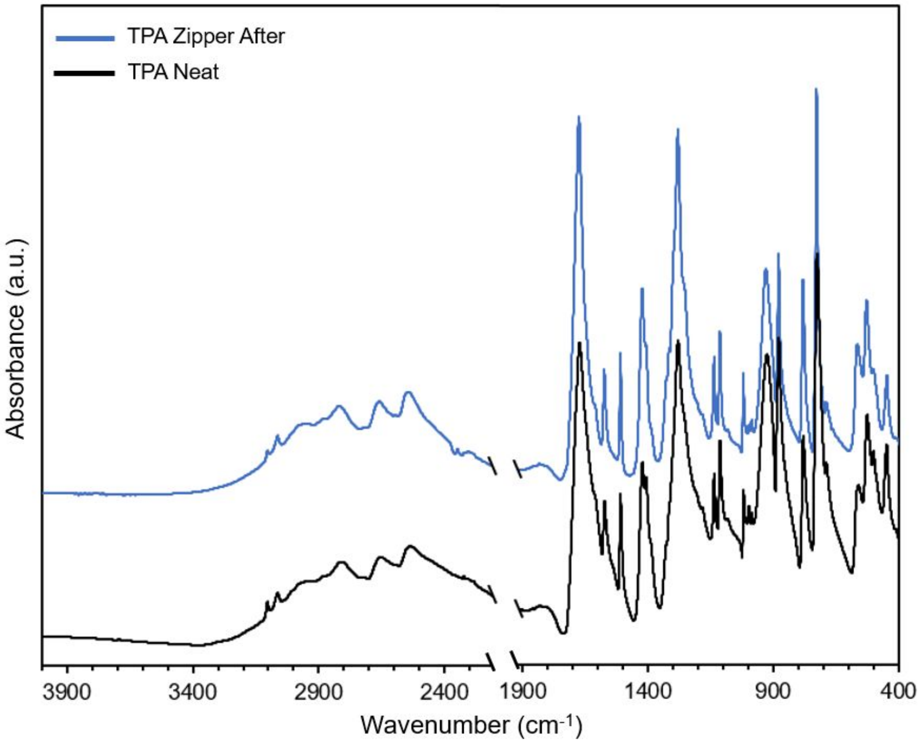

Figure S34. ATR-FTIR (mid-IR) spectra of TPA from zipper (black, bottom), and TPA from after experiment on zipper (blue, top), absorbance spectra are arbitrarily offset for clarity.

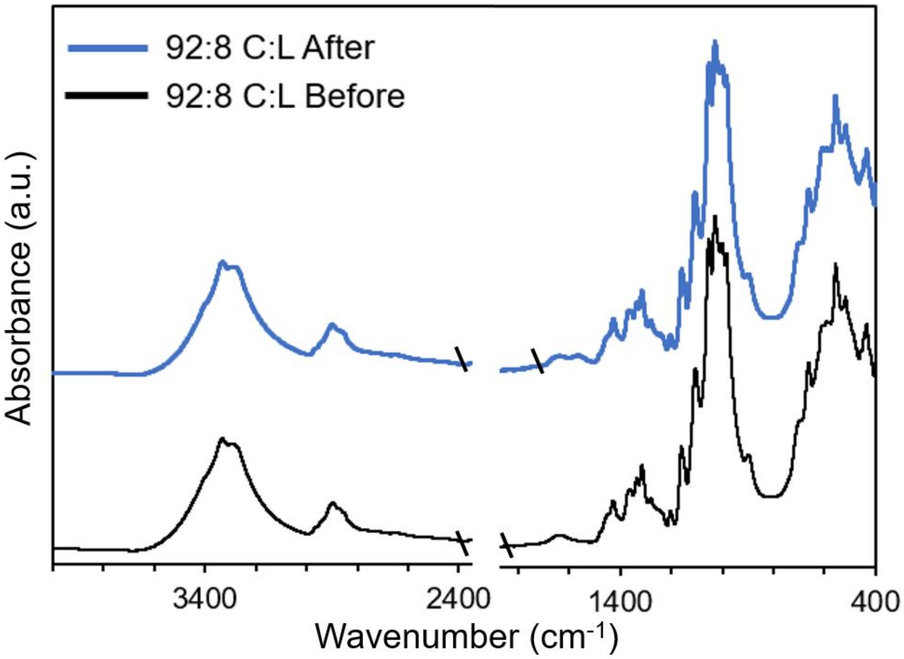

Figure S335. ATR-FTIR (mid-IR) spectra of 92:8 C:L pre-consumer fabric before reaction (black, bottom), and 92:8 C:L pre-consumer fabric after experiment (blue, top),), absorbance spectra are arbitrarily offset for clarity.

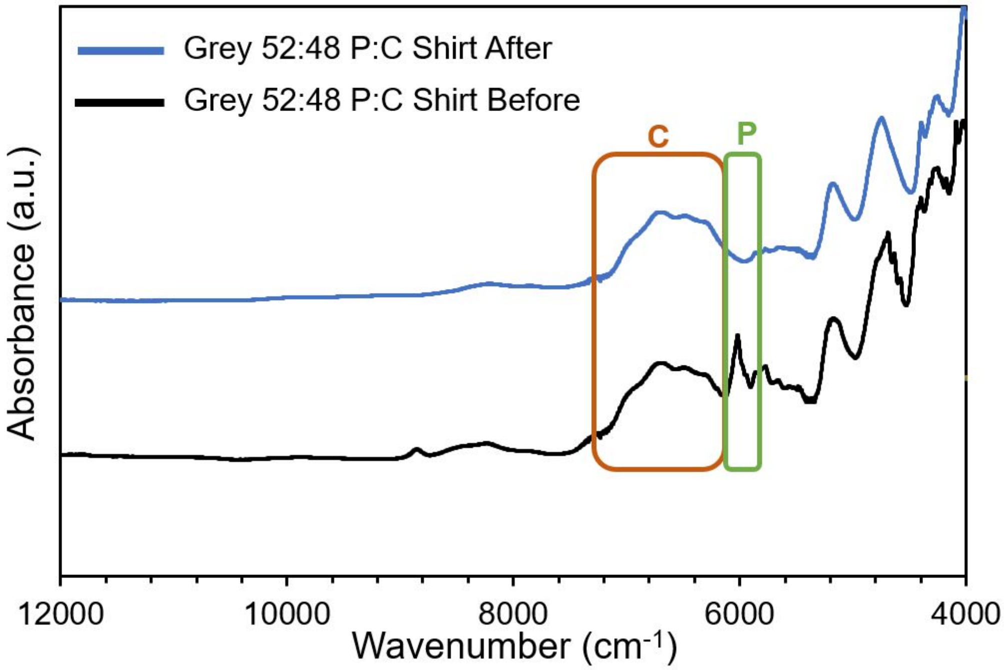

Figure S36. NIR spectra of 52:48 P:C grey post-consumer shirt (black, bottom), and 52:48 P:C grey post-consumer shirt post-reaction (blue, top). Distinct peaks associated with C boxed in orange (left) and peak associate with P boxed in green (right), absorbance spectra are arbitrarily offset for clarity.

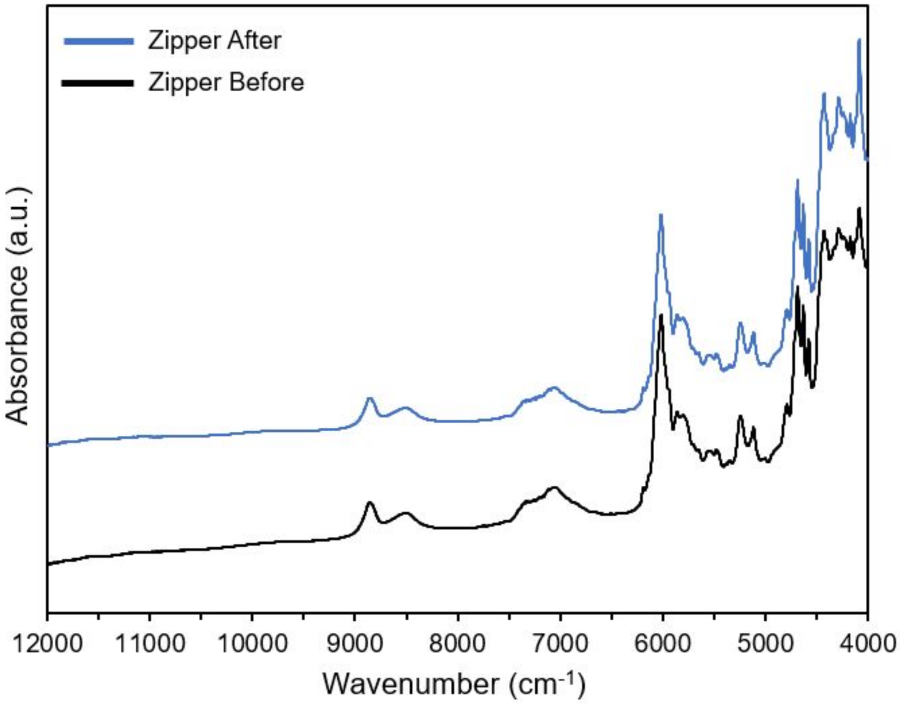

Figure S37. NIR spectra of post-consumer zipper from backpack before (black, bottom), and post-consumer zipper from backpack post-reaction (blue, top), absorbance spectra are arbitrarily offset for clarity.

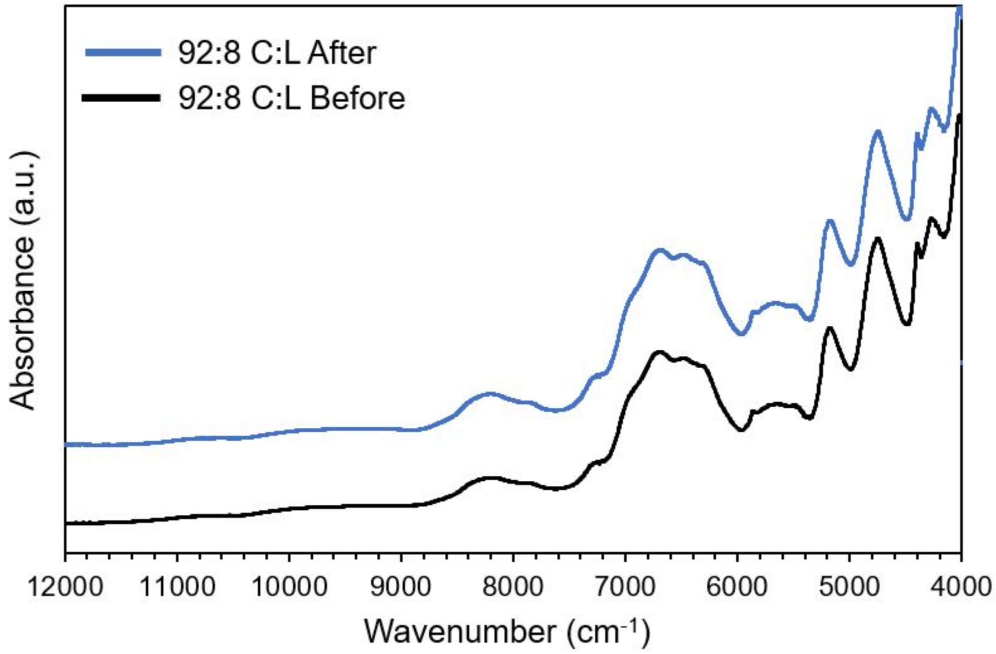

Figure S38. NIR spectra of 92:8 C:L pre-consumer material before reaction (black, bottom), and 92:8 C:L pre-consumer material post-reaction (blue, top), absorbance spectra are arbitrarily offset for clarity.

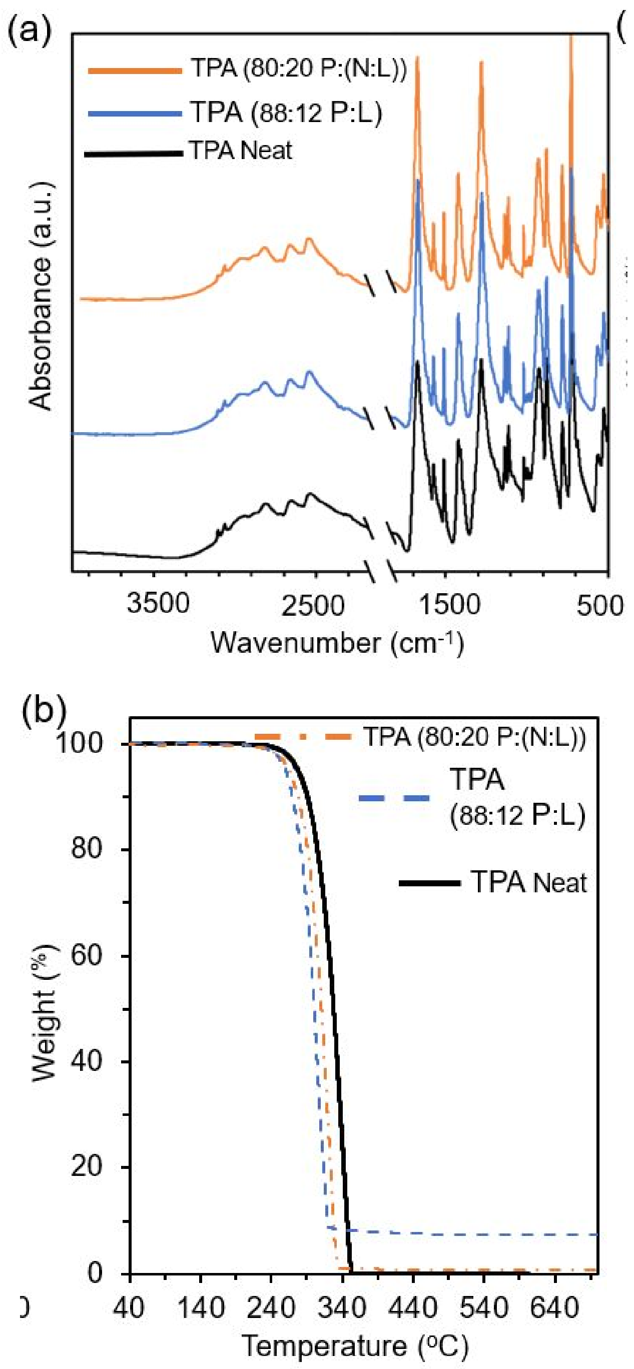

Figure S39. Characterization of TPA produced from pre-consumer materials containing N and L. (a) MID-IR spectra and (b) TGA with TPA from distributer (black), TPA from 88:12 P:L (blue), and TPA from a 80:20 P:(N:L) (orange). Discussed alongside **Figure 8.**

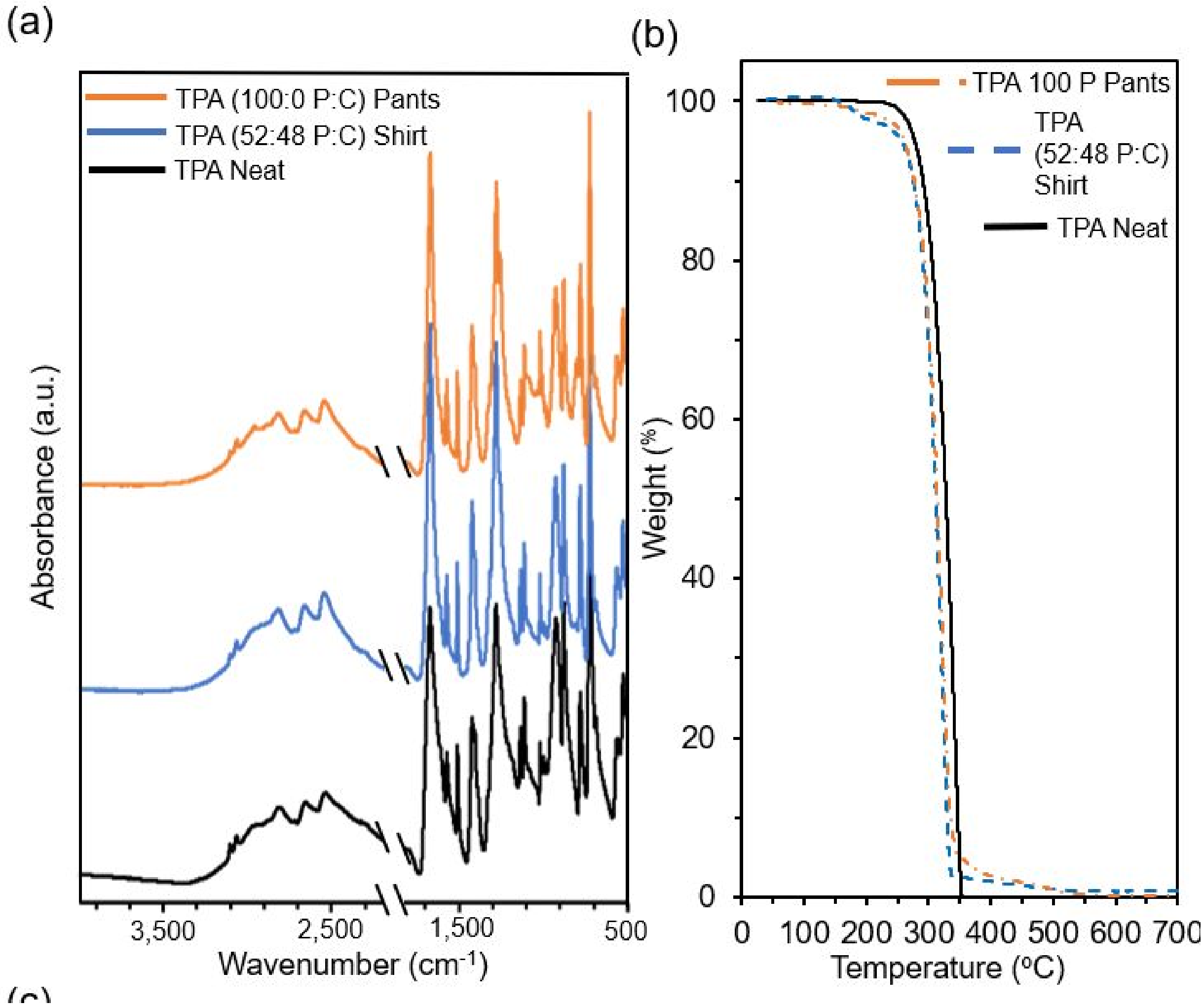

Figure S40. Characterization of post-consumer material's post-reaction. (a) MID-IR spectra and (b) TGA with TPA from distributer (black), TPA from 100 P:C pants (blue), and TPA from 52:48 t-shirt (orange, dashed). Discussed alongside **Figure 9.**

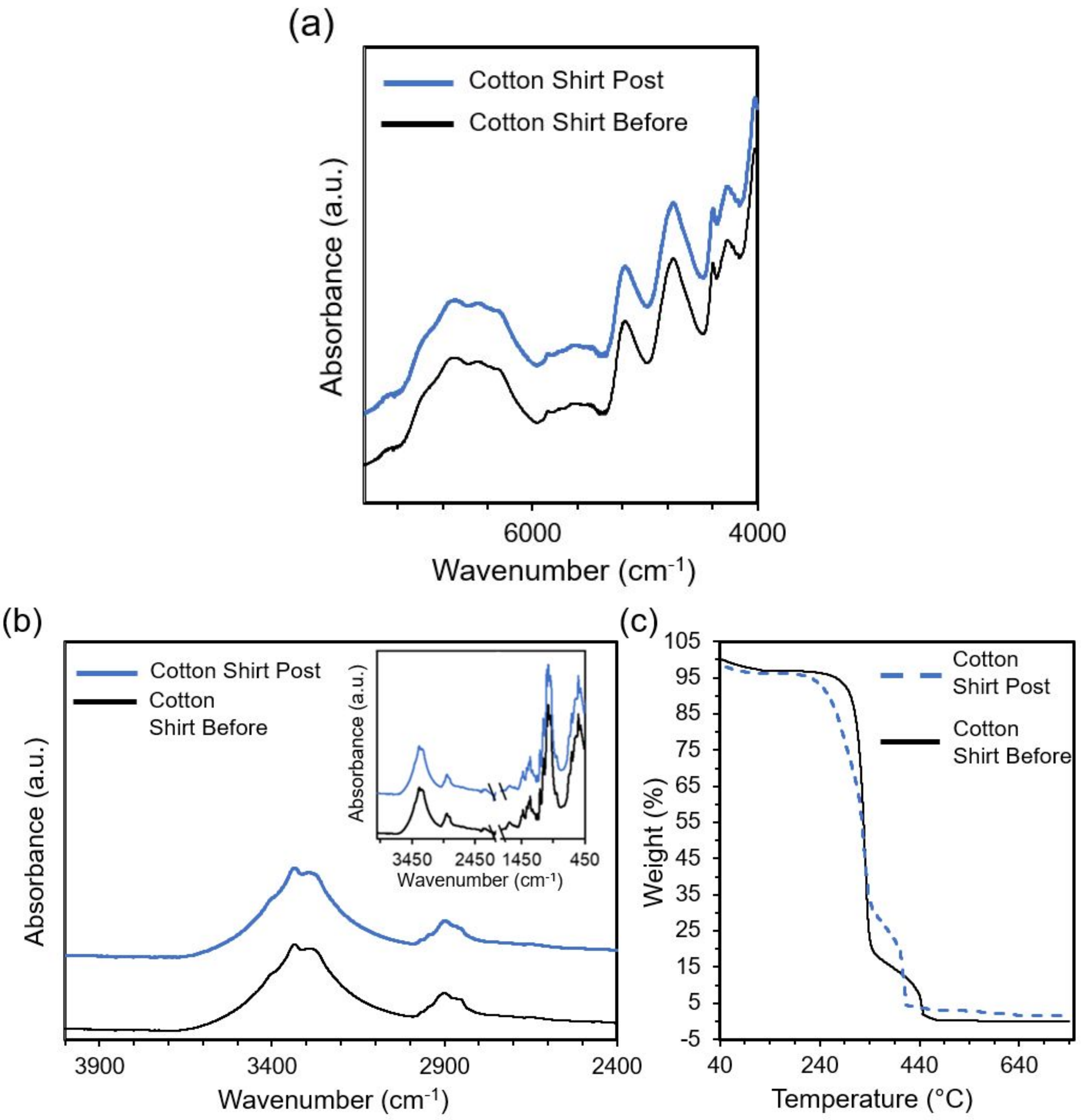

Figure S41. Characterization of post-consumer cotton shirt pre- (black) and cotton shirt post-reaction (blue), (a) NIR, (b) MID-IR spectra (full spectra inlet), and (c) TGA.

Section 3: Data Availability

Available as a NIST Data Repository: https://doi:10.18434/mds2-3727

References:

(1) *Forensic Fiber Reference Collection.* Microtrace. https://www.microtrace.com/service/forensic-fiber-reference-collection/ (accessed 2025-01-08).

(2) Palme, A.; Peterson, A.; De La Motte, H.; Theliander, H.; Brelid, H. Development of an Efficient Route for Combined Recycling of PET and Cotton from Mixed Fabrics. *Text Cloth Sustain* **2017**, *3* (1), 4. https://doi.org/10.1186/s40689-017-0026-9.

(3) Kosmidis, V. A.; Achilias, D. S.; Karayannidis, G. P. Poly(Ethylene Terephthalate) Recycling and Recovery of Pure Terephthalic Acid. Kinetics of a Phase Transfer Catalyzed Alkaline Hydrolysis. *Macromolecular Materials and Engineering* **2001**, *286* (10), 640–647. https://doi.org/10.1002/1439-2054(20011001)286:10<640::AID-MAME640>3.0.CO;2-1.

(4) López-Fonseca, R.; González-Marcos, M. P.; González-Velasco, J. R.; Gutiérrez-Ortiz, J. I. A Kinetic Study of the Depolymerisation of Poly(Ethylene Terephthalate) by Phase Transfer Catalysed Alkaline Hydrolysis. *Journal of Chemical Technology & Biotechnology* **2009**, *84* (1), 92–99. https://doi.org/10.1002/jctb.2011.

(5) Velasco, J. R. G. A Shrinking Core Model for the Alkaline Hydrolysis of PET Assisted by Tributylhexadecylphosphonium Bromide. *Chemical Engineering Journal* **2009**.

(6) Effect of Quaternary Ammonium Salt as a Phase Transfer Catalyst for the Microwave Depolymerization of Polyethylene Terephthalate Waste Bottles. *ResearchGate* **2024**. https://doi.org/10.1016/j.cej.2012.03.081.
